# The Effectiveness of Natural Diarylheptanoids against *Trypanosoma cruzi*: Cytotoxicity, Ultrastructural Alterations and Molecular Modeling Studies

**DOI:** 10.1371/journal.pone.0162926

**Published:** 2016-09-22

**Authors:** Vitor Sueth-Santiago, Julliane de B. B. Moraes, Eliomara Sousa Sobral Alves, Marcos André Vannier-Santos, Célio G. Freire-de-Lima, Rosane N. Castro, Gustavo Peron Mendes-Silva, Catarina de Nigris Del Cistia, Luma Godoy Magalhães, Adriano Defini Andricopulo, Carlos Mauricio R. Sant´Anna, Debora Decoté-Ricardo, Marco Edilson Freire de Lima

**Affiliations:** 1 Universidade Federal Rural do Rio de Janeiro, Instituto de Ciências Exatas, Departamento de Química, BR 465, Km 07, CEP: 23.890-000, Seropédica, RJ, Brazil; 2 Universidade Federal Rural do Rio de Janeiro, Instituto de Veterinária, Departamento de Microbiologia e Imunologia Veterinária, BR 465, Km 07, CEP: 23.890-000, Seropédica, RJ, Brazil; 3 Laboratório de Biologia Parasitária, Centro de Pesquisas Gonçalo Moniz (CPqGM-Fiocruz), Rua Waldemar Falcão, 121, Candeal, CEP: 40.296-710, Salvador, BA, Brazil; 4 Universidade Federal do Rio de Janeiro, Instituto de Biofísica Carlos Chagas Filho, Ilha do Fundão, Cidade Universitária, CEP: 21.941-902, Rio de Janeiro, RJ, Brazil; 5 Universidade Federal Rural do Rio de Janeiro, Instituto de Ciências Exatas, Departamento de Matemática, BR 465, Km 07, CEP: 23.890-000, Seropédica, RJ, Brazil; 6 Laboratório de Química Medicinal e Computacional, Centro de Pesquisa e Inovação em Biodiversidade e Fármacos, Instituto de Física de São Carlos, Universidade de São Paulo, CP 396, CEP: 13.560-970, São Carlos, SP, Brazil; Ain Shams University, EGYPT

## Abstract

Curcumin (CUR) is the major constituent of the rhizomes of *Curcuma longa* and has been widely investigated for its chemotherapeutic properties. The well-known activity of CUR against *Leishmania sp*., *Trypanosoma brucei* and *Plasmodium falciparum* led us to investigate its activity against *Trypanosoma cruzi*. In this work, we tested the cytotoxic effects of CUR and other natural curcuminoids on different forms of *T*. *cruzi*, as well as the ultrastructural changes induced in epimastigote form of the parasite. CUR was verified as the curcuminoid with more significant trypanocidal properties (IC_50_ 10.13 μM on epimastigotes). Demethoxycurcumin (DMC) was equipotent to CUR (IC_50_ 11.07 μM), but bisdemethoxycurcumin (BDMC) was less active (IC_50_ 45.33 μM) and cyclocurcumin (CC) was inactive. In the experiment with infected murine peritoneal macrophages all diarylheptanoids were more active than the control in the inhibition of the trypomastigotes release**.** The electron microscopy images showed ultrastructural changes associated with the cytoskeleton of the parasite, indicating tubulin as possible target of CUR in *T*. *cruzi*. The results obtained by flow cytometry analysis of DNA content of the parasites treated with natural curcuminoids suggested a mechanism of action on microtubules related to the paclitaxel`s mode of action. To better understand the mechanism of action highlighted by electron microscopy and flow cytometry experiments we performed the molecular docking of natural curcuminoids on tubulin of *T*. *cruzi* in a homology model and the results obtained showed that the observed interactions are in accordance with the IC_50_ values found, since there CUR and DMC perform similar interactions at the binding site on tubulin while BDMC do not realize a hydrogen bond with Lys163 residue due to the absence of methoxyl groups. These results indicate that trypanocidal properties of CUR may be related to the cytoskeletal alterations.

## Introduction

**Fig 1:**
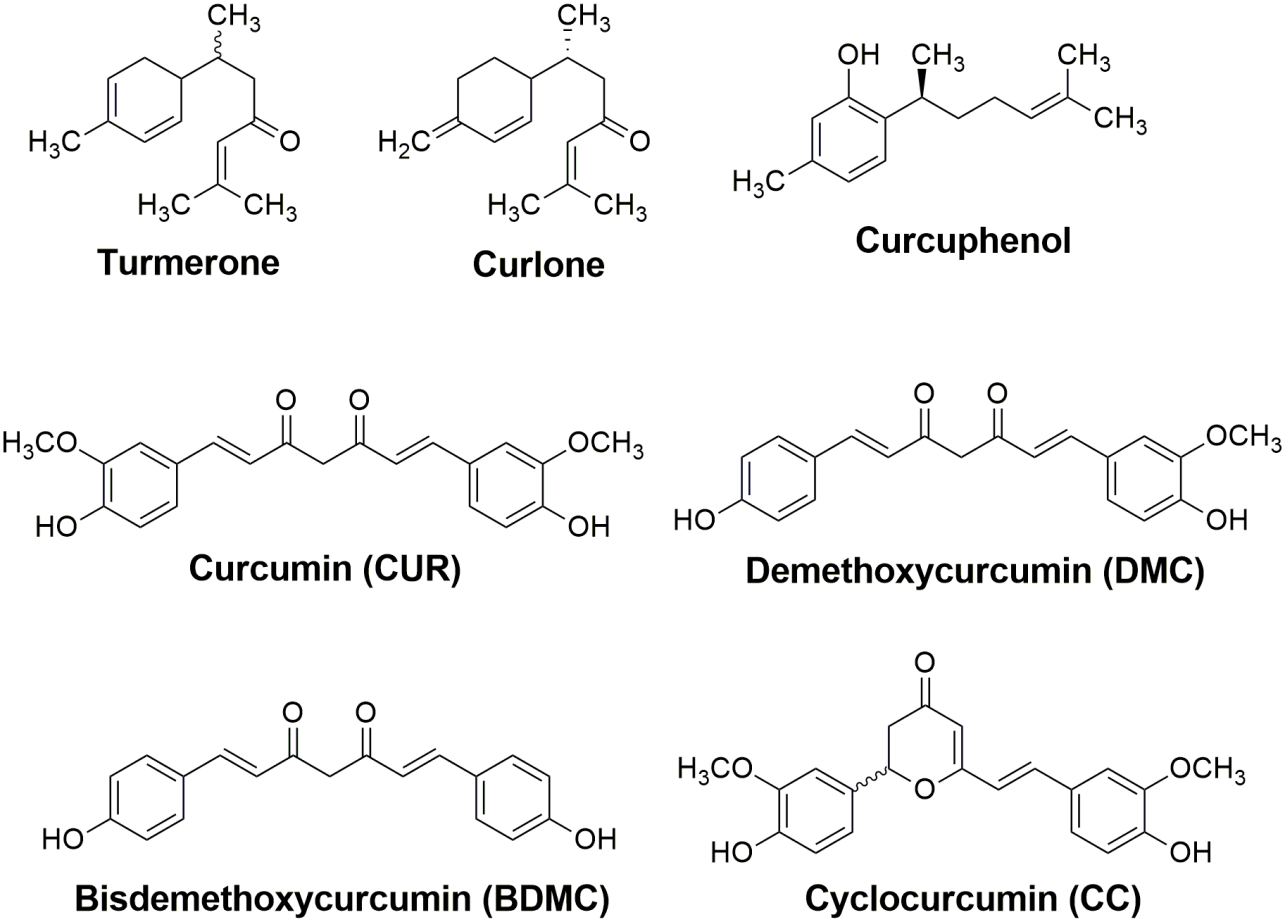
Structures of the main chemical constituents from *Curcuma longa*.

*Curcuma longa* is a plant belonging to the Zingiberacea family, which is endemic in South and Southeast of Asian Continent, being cultivated in many countries worldwide [1]. The dried and powdered roots of *C*. *longa*, known as Turmeric, have many uses as textile dyes, as herbal medicines, or as food products, *e*.*g*. in sauces such as curry; which points the great relevance of the knowledge about the biological properties of its chemical components. The main special metabolites present in *C*. *longa* belong to the sesquiterpene and diarylheptanoid classes. Turmerone, curcuphenol and curlone (Fig 1) are the main sesquiterpenes in turmeric, with the concentrations of 30.0, 10.6 and 10,0% in the plant rhizomes, respectively [2]. Turmerone, one of the components responsible for the aroma of the *C*. *longa* essential oil, shows important biological properties such as inhibition of platelet aggregation [3] and antidiabetic [4]. Another important class of special metabolites present in *C*. *longa* are the diarylheptanoids, structurally related to curcumin (CUR), which is the most representative example of this group [5]. Besides CUR, two other minor diarylheptanoids are found in *C*. *longa*, demethoxycurcumin (DMC) and bisdemethoxycurcumin (BDMC, Fig 1). It’s also described in the literature the detection of a curcuminoid non-diarylheptanoid (cyclocurcumin, CC, Fig1) in the crude mixture [6]. CUR was originally isolated in 1818 by Vogel, and its chemical structure was elucidated in 1910 by Lampe [7]. Certainly, the majority of the biological activities assigned to turmeric since ancient times are due to natural diarylheptanoids [8]. The α,β-unsaturated dihydropyranone moiety present in CC is formed through a Michael-type addition cyclization of curcumin.

The structural similarity between the three diarylheptanoids present in *C*. *longa* compounds gives them similar physico-chemical properties, which hampers the discrimination of each component during their isolation from the biological matrix. Due to this fact, the commercial curcumin is actually a mixture of curcuminoids enriched in CUR (75–81%), but containing also DMC (15–19%) and BDMC (2–6%). The isolation of these mixed components may be performed in several ways, such as solvent-based extractions, percolations and extractions assisted by supercritical fluid and microwave [9].

CUR is associated with the modulation of several biological activities such as anti-inflammatory effects, hepatoprotective, antioxidant and notorious chemotherapeutic properties against bacteria, fungi, protozoa and tumor cells [1,2,5]. In the application of CUR in the treatment of parasitic diseases, it should be highlighted the possibility of its use in the chemotherapy of neglected diseases. These illnesses are so called due to low investment from the pharmaceutical industry and public health systems in research and development of drug candidates that could lead to its cure. The biggest problem of this issue is the fact that the neglected diseases such as malaria, leishmaniasis, trypanosomiasis and other parasitic maladies, together with tuberculosis, had only 0.1% of global investment in health research while these diseases together contribute about 5% of the global disease burden [10]. Despite their impact on population health, there are no effective drugs. Chagas disease, for example, is an illness with relevance in the American continent, being endemic in most countries on its Southern part. The etiologic agent of Chagas disease is *Trypanosoma cruzi* (Trypanosomatidae) a flagellated protozoan circulating the peripheral blood of their vertebrate host and multiply within cells of vital organs (*e*.*g*. heart and liver), causing extensive cell death and tissue damage [11].

In the development of new drugs for the treatment of parasitic infections selectivity is a very important parameter. The toxicity of the new chemical entities must be evaluated both against the cells of the disease`s etiologic agent as well as the host cells [12]. CUR is, *a priori*, innocuous to the human organism since it is present in considerable amounts in human diet, thus it is possible to ensure a relatively safe administration. Previous studies carried out by our group have shown that CUR is active against *Leishmania amazonensis*, possibly due to its structural similarity with pentamidine, a drug employed in the treatment of leishmaniasis [13]. Although Nagajyothi and co-workers had shown in a previous study that commercial CUR was able to provide protection against infection of *T*. *cruzi* both *in vitro* and *in vivo*, the authors performed their work using a traded curcumin labeled as 65% pure by HPLC (probably due to the presence of minority curcuminoids). Due to this fact, the results obtained by the authors cannot be attributed only to curcumin, as reported in the work [14]. Furthermore, Nose and co-workers also found that CUR showed cytotoxic activity *in vitro* against *Trypanosoma brucei* [15]. These previous results reported in the literature encouraged us to investigate more accurately the activity of CUR as well as the other minor natural diarylheptanoids (DMC, BDMC and CC), each in it`s pure form (purity > 95% assessed by HPLC-DAD), against the different evolutionary forms of *T*. *cruzi*.

## Results and Discussion

### Chemistry

A mixture of curcuminoids present in commercially available curcumin after chromatographic separation by TLC and HPLC (as shown in S1 Fig) had each component isolated, purified and fully characterized. This mixture present in the commercial material is not resolved by thin layer chromatography (TLC) using hexanes and ethyl acetate as eluent, but it is possible to identify three spots when methanol and dichloromethane (2:98) are used. Several studies in the literature report the use of commercial curcumin in biological assessments without prior purification, so the results described in these works involve actually the evaluation of the three curcuminoids as a mixture (CUR, DMC and BDMC, Fig 1).

The recrystallization of the mixture in methanol yielded pure CUR, followed by separation of the other two minor components present in the mother liquor (DMC and BDMC) by column chromatography on silica-gel. Due to the fact that CC (Fig 1) occurs in very small amounts in natural matrix, there are no previous studies on biological evaluation of this molecule. So as CC was not detected in the mixture used in this study, it was synthesized in order to determine the influence of this scaffold modification in the trypanocidal properties of the three diarylheptanoids.

#### Isolation of the curcuminoids

Commercial curcumin was subjected to recrystallization in order to isolate pure curcumin. CUR was obtained in suitable purity after successive recrystallizations from a mixture of methanol/water (7:3). The mother-liquor enriched in DMC and BDMC was then subjected to separation by column chromatography on silica-gel. The silica used was previously adsorbed with NaH_2_PO_4_ and activated at 200°C overnight to minimize the oxidation of curcuminoids [16]. Dichloromethane was used as eluent and the fractions collected were grouped according to their chromatographic profiles. By this way CUR, DMC and BDMC were obtained in suitable amounts for their characterization by spectroscopic methods of analysis and in purities over 95% (evaluated by HPLC).

#### Synthesis of symmetrical curcuminoids

**Fig 2:**
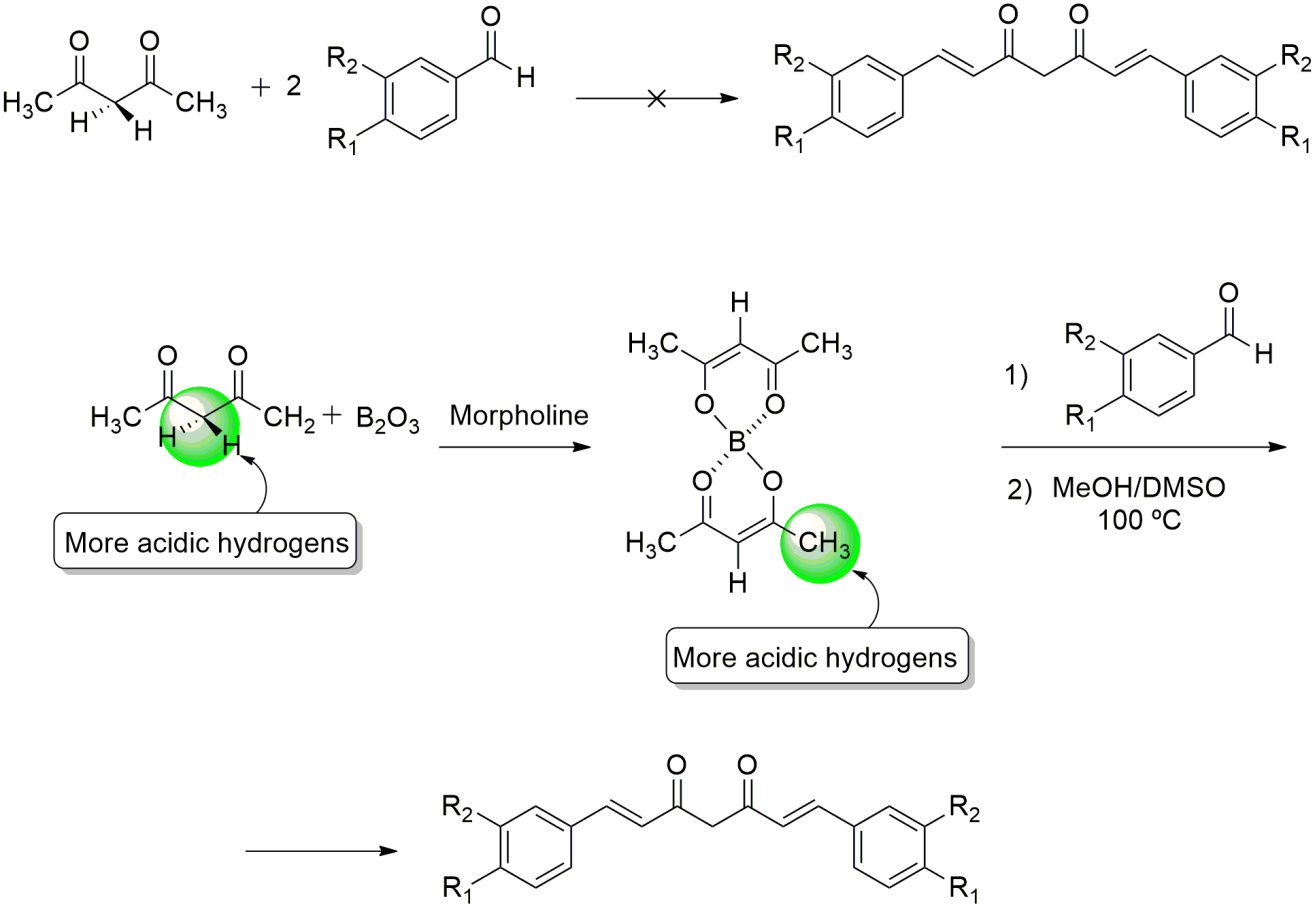
Synthesis of CUR and BDMC via acetylacetone-boron enolate.

Since BDMC was collected in small amounts from the commercial mixture, an experimental procedure was stabilished for the synthesis of symmetrical curcuminoids (CUR and BDMC) in suitable yield and purity [17], allowing access to adequate quantities to carry out all the planned biological assays. The strategy used involved the condensation reaction between acetylacetone (2,4-pentanedione) and two equivalents of the corresponding aldehyde. However, the methylene hydrogens are much more acidic than the methyl ones. To overcome this effect, boric oxide was used, which will form a boron-based enolate, whose more acidic hydrogens are those on the methyl groups of acetylacetone-boron enolate (Fig 2). This enolate will then react with the corresponding aldehyde, and the boron complex can be broken after reflux with methanol and DMSO.

#### Synthesis of cyclocurcumin

**Fig 3:**
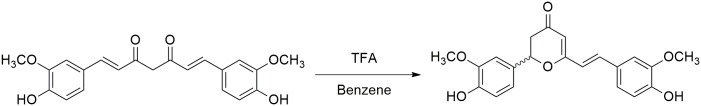
Synthesis of CC by acid catalyzed cyclization of CUR.

Cyclocurcumin is a curcuminoid present in very low concentration in the rhizomes of *C*. *longa* occurring as a racemic mixture. CC was originally described by Kiuchi and co-workers [6], and it is a dihydropyranone that can undergo double bond photoisomerization. The synthesis of CC was performed from pure curcumin in benzene, catalyzed by trifluoracetic acid (Fig 3). This reaction condition was protected from light in order to prevent the isomerization of the final product, which was isolated as a yellow gummy solid in 20% yield after chromatographic purification.

### Biological Assays

The four natural compounds (CUR, DMC, BDMC and CC) were evaluated on the proliferation/survival of *T*. *cruzi* epimastigotes (Dm28c strain) in four different concentrations (100, 50, 25 and 2 μM). The parasites were cultivated for 7 days in BHI (Brain Heart Infusion) medium supplemented with 10% (v/v) fetal bovine serum. Controls were incubated solely and with 0.02% DMSO and the cultures were readily incubated with the natural compounds. Mobile epimastigote forms were considered as viable forms, and were counted on Neubauer chambers using phase contrast microscopy. In this model, all diarylheptanoids CUR, DMC and BDMC were active, except the α,β-unsaturated dihydropyranone CC. CUR, DMC and BDMC showed consistent dose-response curves (as shown in S2 Fig), allowing the calculation of their values of inhibitory concentrations for 50% (IC_50_), as shown in Table 1. CUR was the most active compound, with an IC_50_ = 10.13μM. These results suggest that the diarylheptanoid scaffold (C_6_-C_7_-C_6_) is required for the trypanocidal activity. Similarly, Simon and coworkers [18] showed that CC is inactive in the inhibition of MCF-7 tumor cells proliferation.

**Table 1:** IC_50_ values for epimastigotes of *T*. *cruzi* (Dm28c strain) and LD_50_ for two cell lines.

Compounds	IC_50_ *T*. *cruzi*(μM)	LD_50_ Mø(μM)	LD_50_ Splenocytes(μM)
Curcumin (CUR)	10.13	> 100	> 100
Demethoxycurcumin (DMC)	11.07	> 100	> 100
Bisdemethoxycurcumin (BDMC)	45.33	> 100	> 100
Cyclocurcumin (CC)	> 100	> 100	> 100

The natural products have been subjected to a cytotoxicity assay on two distinct murine cell types. These compounds were tested upon macrophages (Mø), which are host cells during *T*. *cruzi* infection and splenocytes as lymphocytes are highly sensitive to external agents. The maintenance of cell viability at presence of the curcuminoids may indicate their low toxicity to mammalian cells. Peritoneal macrophages harvested from Balb/c mice were treated for 48 hours with concentrations of each test substance (S3 Fig). Splenocyte viability assays were performed in cells obtained from BALB/c mice and grown from a 5x10^5^ cells.mL^-1^ suspension (S4 Fig). For both cell types, viability was assessed by the trypan blue dye exclusion method, because colorimetric methods such as XTT were not suitable due to influence of the curcumin chromophoric properties. The results obtained on the evaluation of CUR, DMC, BDMC and CC against *T*. *cruzi* epimastigotes as well as the results of two host cell types treated with a 100 μM concentration of each compound are summarized in Table 1**.**

**Fig 4:**
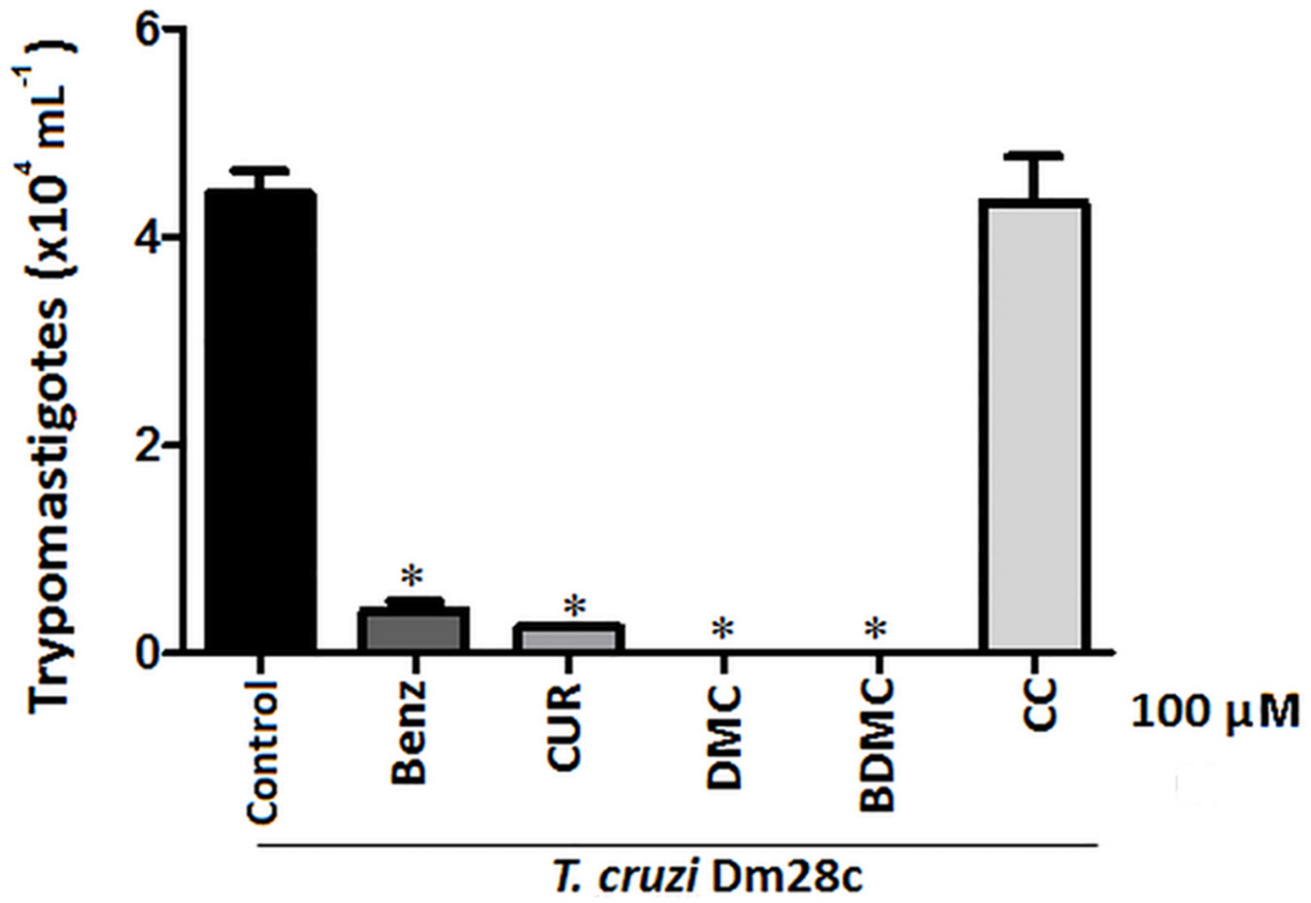
Murine peritoneal macrophages were infected with 10^5^ *T*. *cruzi* trypomastigotes (Dm28c strain). Parasite cultures were treated with 100 μM of benznidazole, CUR, DMC, BDMC and CC. After 7 days, the number of released trypomastigotes in the culture medium were determined. The data shown were obtained from three independent experiments. Cultures were compared using unpaired T-Student test (Graph Pad Prism). *P<0.05.

The toxicity of diarylheptanoid derivatives against epimastigotes of *T*. *cruzi* and the maintenance of cell viability in types of murine cells allowed the realization of an experimental infection *in vitro*. This experiment aims to study the effect of CUR on the amounts of trypomastigotes of *T*. *cruzi* released from infected macrophages *in vitro* (Fig 4). Then, murine peritoneal macrophages were infected with metacyclic trypomastigotes of Dm28c strain. Trypomastigote release in the supernatant was measured before and after 100 μM CUR, DMC, BDMC and CC as well as benznidazole (Benz), used as reference drug for 7 days. The results shown in Fig 4 demonstrate the inhibition of the three natural diarylheptanoid on the release of trypomastigotes from infected mammalian cells, probably by a decrease in the proliferation of intracellular amastigotes, which then do not reach a density that allows them to differentiate into trypomastigotes leading to host cell disruption.

Since the natural diarylheptanoid displayed selective toxicity against the different developmental forms of *T*. *cruzi*, we decided to elucidate the mechanisms underlying the trypanocidal activity of the compounds. Studies about the changes produced in subcellular structures have provided valuable information that enables a detailed understanding about the mechanisms of action involved in antiparasitic agents at cellular level [19].

**Fig 5:**
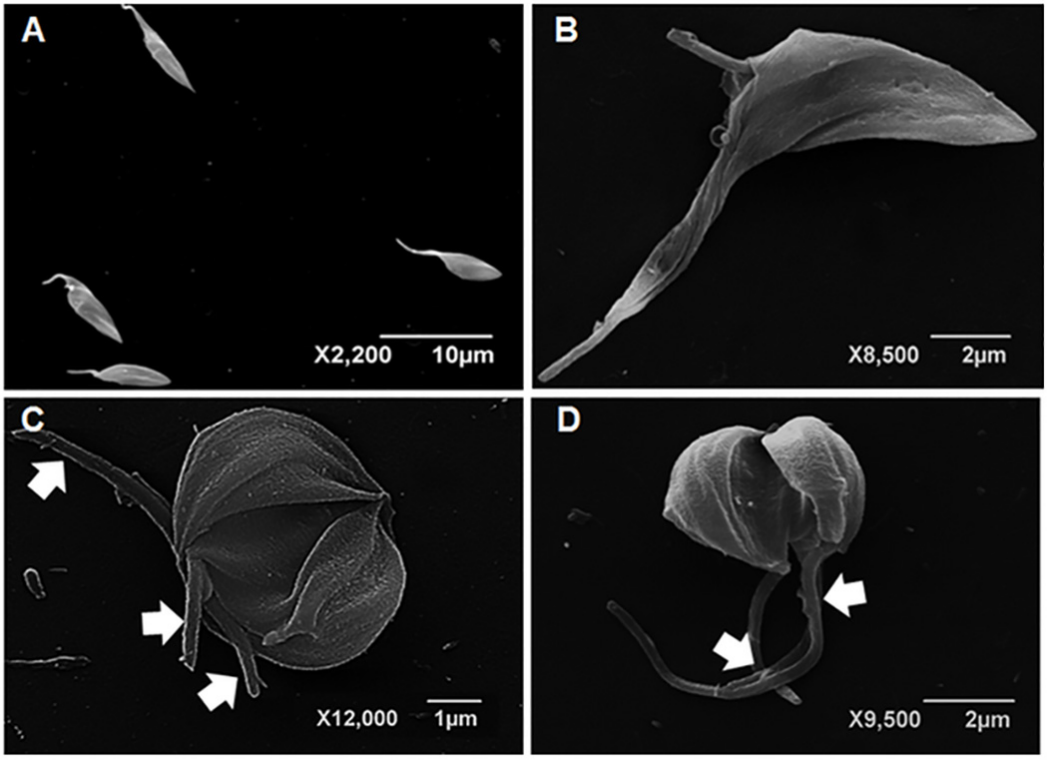
Scanning electron microscopy of *T*. *cruzi* epimastigotes. Untreated control cells (A) displayed the usual elongated morphology with smooth cell surface. Parasites incubated with 10.13 μM curcumin for 24 h (B-D) presented reduced cell volume (B) as well as cell body rounding with multiple longitudinal invaginations involving the anterior portion of the parasite (C,D). Some cells displayed multiple shortened flagella (C, arrows). The protrusion of flagellar membrane was detected (D, arrows).

The action of the compounds on the survival and proliferation of protozoan may be related to its effects on its metabolic pathways such as synthesis of ergosterol and polyamines, interfering with cellular architecture [20–23]. Different microbicides can trigger parasite surface protrusions [19]. The treatment of *T*. *cruzi* with CUR (Fig 5) led to the formation of multiple shortened flagella, which were also previously observed in *T*. *cruzi* incubated with arjunolic acid which interfered with the microtubule function [24]. The rounded cell bodies displaying multiple longitudinal invaginations suggest uncompleted cell division in curcumin-treated *T*. *cruzi* epimastigotes. Similar images were obtained in *Leishmania amazonensis* promastigotes cultured with vinblastine which presented remarkable microtubule alterations [25]. The induced projections (Fig 5) may be associated with the flagellum membrane phospholipid content as reported by Santa-Rita *et al* [26]. The detachment of portions of the flagellar membrane was reported in *Trypanosoma* and *Leishmania* following treatments with sterol biosynthesis inhibitors [21], phorbol esters [27,28]. Fibronectin [29] and cationized ferritin-induced shedding [30] are also associated with flagellar membrane displacement. The flagellar and cell body alterations may be due at least in part to the action of CUR and derivatives upon cytoskeleton organization. In this regard CUR was reported to produce microtubules disorganization in cancer cells [31–33] and in *Plasmodium falciparum* [34]. Similar flagellar membrane detachment was reported after parasite incubation with semi-purified subfraction obtained of *Anthemis tinctoria* [35]. The blebbing and shedding of parasite membranes must also be kept in mind since these microsomes or vesicles may bind to non-infected cells, which are recognized by anti-*T*. *cruzi* antibodies [36]. Interestingly the extracellular vesicles also known as exosomes may play pivotal roles in protozoal parasitic infections, taking part in *T*. *cruzi* immune evasion and chagasic cardiomyopathy [37].

**Fig 6:**
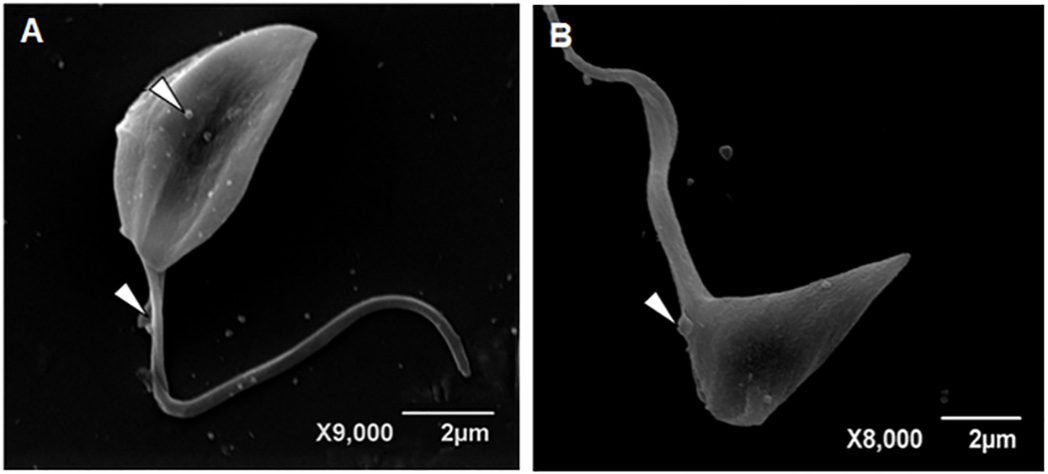
Scanning Electron Microscopy of *T*. *cruzi* epimastigotes. Parasite were cultured with 11.07 μM DMC for 24 h (A, B). There was apparent reduction of cell volume and membrane protrusions over cell body and flagellar surfaces (arrowheads).

**Fig 7:**
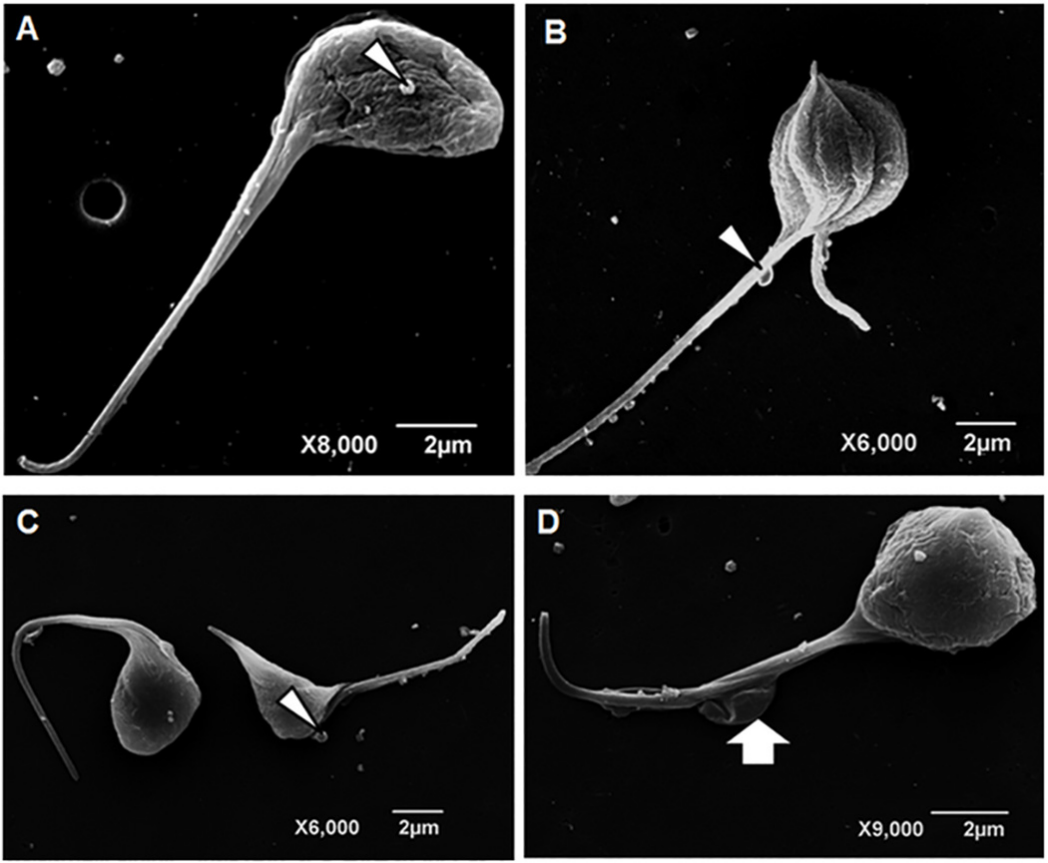
Scanning electron microscopy of *T*. *cruzi* epimastigotes. Parasites were cultured for 72 h with 11.07 μM DMC (A e B) and 45.33 μM BDMC (C, D). Parasite cell bodies displayed wavy (A) or grooved (B) surfaces and reduced cell volumes (A-D). Surface blebbing was observed on both cell body and flagellar membranes (A-C). Large portions of protruded flagellar membrane were observed (D, arrow).

Parasites treated with the curcuminoids DMC and BDMC, showed an apparent decrease in cell volume (Figs 6 and 7), presumably due to loss of cytoplasmic contents, possibly promoted by cytoskeleton disorganization.

**Fig 8:**
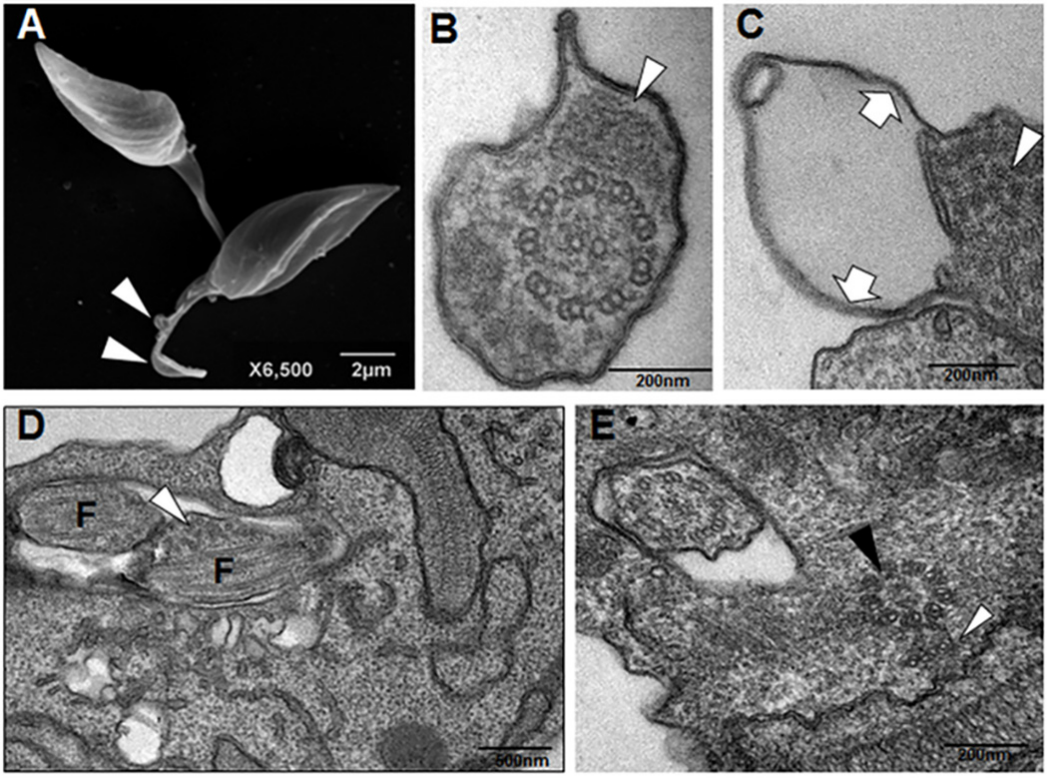
Scanning electron microscopy showing *T*. *cruzi* epimastigotes incubated with curcumin for 48 h (A) and transmission electron microscopy of parasites treated for 72 h (B-E). Curcumin at 10.13 μM induced the formation of flagellar protrusions (A, arrowheads). The paraflagellar rod (B White arrowhead) is partially disorganized (B, black arrowhead). (E). Besides intraflagellar disorganization (C, arrowhead) and curcumin-treated cells displayed flagellar membrane detachment (C, arrows). Such disorganization was observed in flagella (F) within the flagellar pocket lumen (D, arrowhead) and may be associated to basal body (E, black arrowhead) alterations, which included ectopic microtubules duplets (white arrowhead).

CUR-treated epimastigotes displayed disorganized and/or displaced microtubules both within flagella in the basal body vicinity (Fig 8) and the membrane protrusions were devoid of cytoplasmic core, showing displacement from microtubules. These alterations point out to an impaired cytoskeleton functioning including the membrane connection.

Since the detachment of the flagellar membrane was reported in *T*. *cruzi* and *L*. *amazonensis* treated with sterol biosynthesis inhibitors, the results obtained in microscopy study led us to investigate the sterol content of epimastigotes treated with CUR. Sterol 14*α*-demethylase (CYP51) is the enzyme involved in conversion of lanosterol to ergosterol in the parasite cell by oxidative removal of the sterol 14*α*-methyl group, being a key step in the biosynthesis of cell membrane 14 α-demethylated sterols and consequently in the maintenance of the cell viability [38]. Thus, this enzyme is a promising target for chemotherapy of Chagas disease [39]. The method reported by Pinto and co-workers [40] for steroid determination on fungi was adapted to *T*. *cruzi* epimastigotes. After saponification followed by *n*-hexane extraction of a *T*. *cruzi* epimastigote pellet cultivated in the presence of two different concentrations of CUR, the organic layer was concentrated and submitted to RP-HPLC analysis (as shown in S5 Fig). Although the observations on the electron microscopy has indicated a possible inhibition of sterol biosynthesis that could justify the cytotoxic activity of CUR against *T*. *cruzi*, the chromatographic analysis of lanosterol/ergosterol ratio showed no significant difference between treated and untreated control parasites. Furthermore, the analysis of sterol content of the positive control, in which the parasites were treated with posaconazole, a known *T*. *cruzi* 14 α-demethylase inhibitor, showed a significant increase in the ratio lanosterol/ergosterol (S5 Fig). These results indicate that another mechanism of action must be associated with the trypanocidal activity of this natural product. It seems that in both treatments the parasite membrane-cytoskeleton linkage has been compromised.

**Fig 9:**
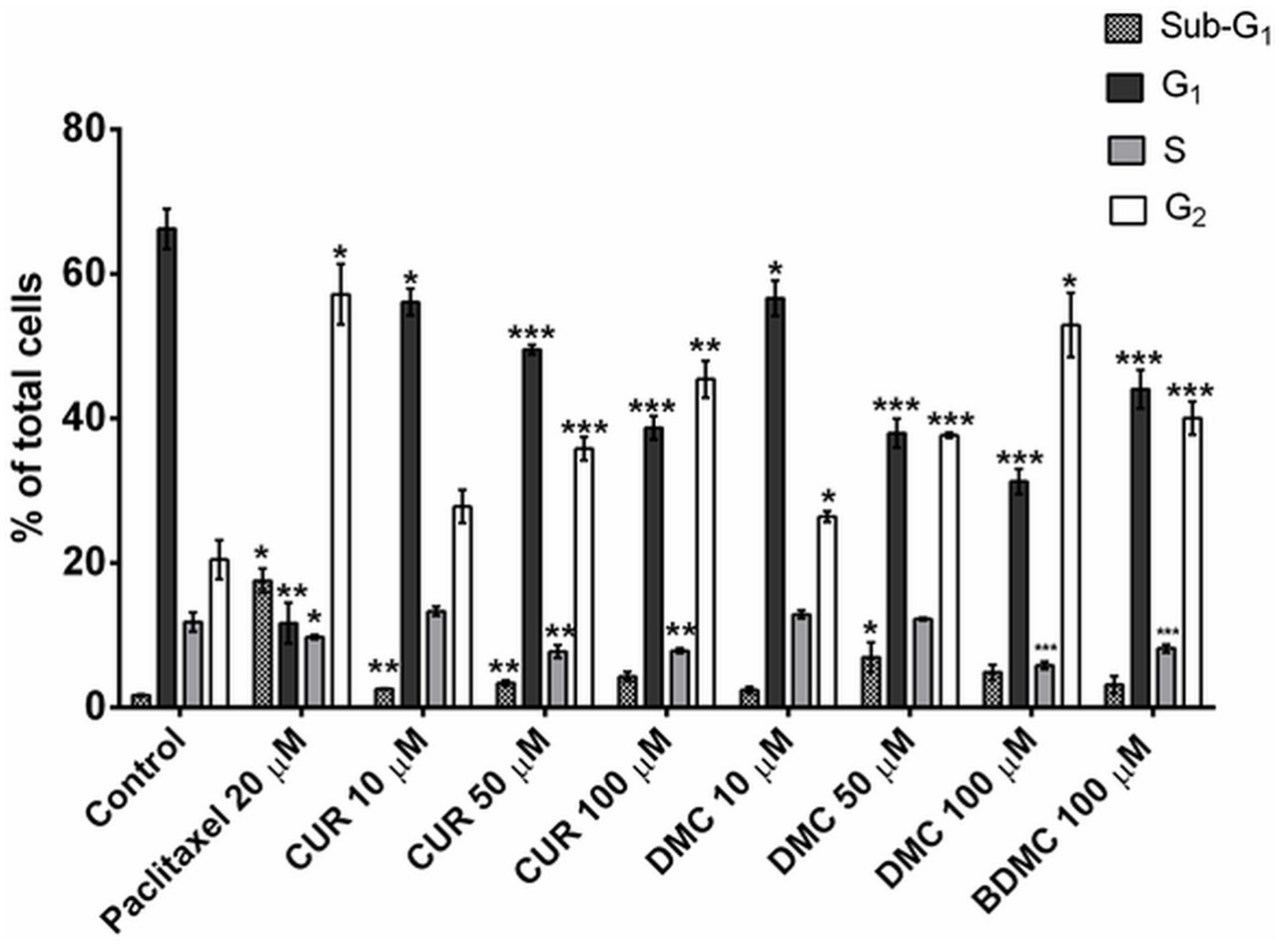
Percentage of cells in different phases of cell cycle. Cells were treated with CUR and DMC at 10, 50 and 100 μM concentrations and with 100 μM BDMC. Paclitaxel (20 μM) was employed as positive control, and DMSO 0.02% as negative control. Results are the mean of the triplicates with standard deviation represented by error bars. Statistical significance relative to control * *P*<0.05, ** *P*<0.01 and *** *P*<0.001. The data shown were obtained from three independent experiments.

The flow cytometry analysis of DNA content of the parasites showed that untreated control exhibited a typical histogram for a normal cell population, with most cells in the G_1_ phase and a smaller population in G_2_ phase (as seen in S6 Fig). Otherwise, treatment with CUR and DMC resulted in an accumulation of cells in the G_2_/M phase, with reduction of the number of cells in G_1_ phase, in a concentration dependent manner (Fig 9). The cultures treated with CUR, DMC and BDMC at a concentration of 100 μM led to an accumulation of, respectively, 45%, 52% and 40% of cells in the G_2_ phase, while in the non-treated cells, G_2_ population was 20%. The ability to arrest cells in the G_2_/M phase is a characteristic of microtubule-interacting agents [41]. For this reason, we used paclitaxel as positive control, a known microtubule drug [42] that is active against Trypanosomatidae organisms [43,44]. Consistently with its action on microtubules, paclitaxel promoted an accumulation of 57% of cells in the G_2_ phase (Fig 9). Thus, the effects of curcuminoids in the cell cycle can be explained, at least partially, by the disturbance of microtubules, in agreement with the mechanism of interaction with tubulin proposed by the electron microscopy experiments. The arising of sub-G_1_ peaks possibly correspond to apoptotic cells, what is more prominent in paclitaxel than in the curcuminoids treated cultures and the small sub-G_1_populations indicate that curcuminoids promote low apoptosis (Fig 9).

### Tubulin Homology Model

The *β*-*α*-*β-*heterotrimer was constructed from *T*. *cruzi α*- and *β*-tubulin subunits, using as templates the B, C and D chains, respectively, from the tubulin-epothilone A complex deposited in the PDB (4I50 code) [42]. Table 2 presents details about the models of tubulin:PDB code of the template, sequence identity, GMQE (Global Model Quality Estimation), which is a quality estimation that is expressed as a number between zero and one, reflecting the expected accuracy of a model built with that alignment and template [45], QMEAN4, which is a composite scoring function for the estimation of the global and local model quality, consisting of four structural descriptors [46], and the quaternary structure information of the models.

**Table 2:** *T*. *cruzi* tubulin chains *models*.

Tubulin Chain	PDB code of template (chain)	Sequence identity (%)	GMQE	QMEAN4
*β*-tubulin	4I50 (B)	85.27	0.98	-1.61
*α*-tubulin	4I50 (C)	84.04	0.94	-1.38
*β*-tubulin	4I50 (D)	84.74	0.93	-1.47

The heterotrimer was constructed with the Swiss PDB-Viewer 4.01 program by superposition of each adequate monomeric model of *T*. *cruzi* tubulin subunit with the B, C and D chains of its tetrameric template, 4I50. This trimeric model of *T*. *cruzi* tubulin, after energy minimization with the GROMOS96forcefield [47], available in Swiss PDB-Viewer 4.01, presented a very low RMSD value when superimposed to 4I50, 0.36 Å, and was used for the subsequent docking study.

#### Molecular docking

Redocking experiments were successful for both colchicine and vinblastine with the four scoring functions available in GOLD 5.2. The default score function, ChemPLP [48], was chosen for the subsequent docking procedure with CUR, DMC and BDMC because this fitness score function is claimed to be generally more effective than the other ones for both pose prediction and virtual screening. With ChemPLP, the RMSD between the best ranked docking poses and the corresponding co-crystal structures for vinblastine and colchicine were 0.98 and 0.21 Å, respectively, indicating the very good quality of the ligand pose prediction with this score function.

**Fig 10:**
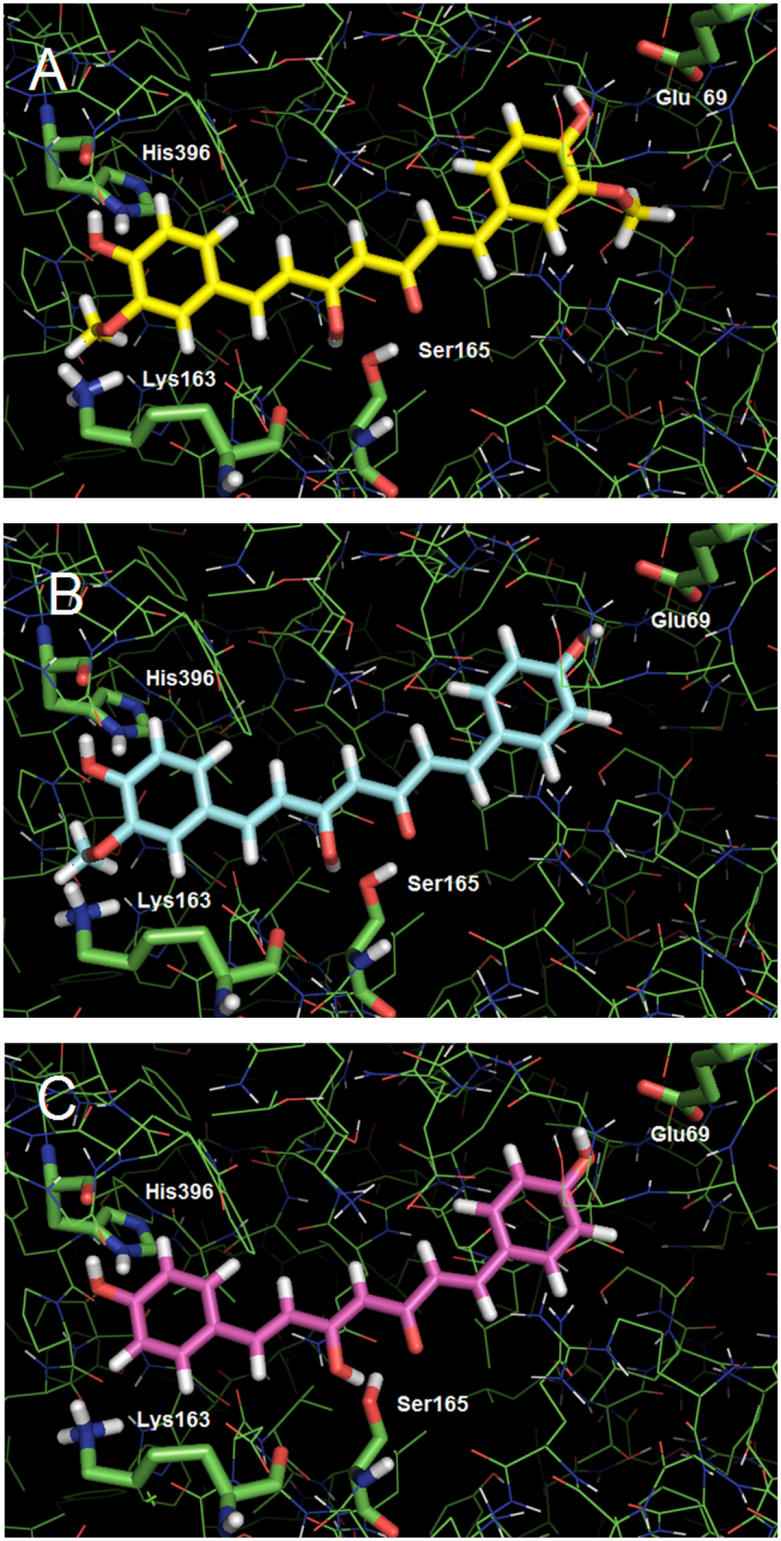
Superposition of best qualified poses of CUR (Entry A), DMC (Entry B) and BDMC (Entry C) after docking into *T*. *cruzi* tubulin. Hydrogen atoms were omitted for clarity; carbon atoms in green: tubulin; yellow: CUR; cyan: DMC; magenta: BDMC. (image generated with PyMOL, DeLano Scientific LLC)

As expected from the results previously obtained by Chakraborti and collaborators [33] with the *B*. *taurus* tubulin, all three curcumin derivatives were able to effectively dock into the *T*. *cruzi* tubulin at the “curcumin binding site” located in the interface between the β- and α-tubulin subunits. The corresponding scores of the best poses correlated quite well with the observed IC_50_ data, indicating a similarity of interactions for CUR and DMC, and a poorer interaction profile for BDMC (Table 1). Analysis of the resulting poses shows the reason for the different profiles: although the binding poses of the three derivatives are very similar, CUR and DMC establish hydrogen bonds with exactly the same amino acid residues, whereas BDMC is not able to make a hydrogen bond with the side chain of Lys163 because of the absence of the methoxy groups (Fig 10C). The absence of one methoxy group in DMC makes no difference for the interaction profile, because only one of the methoxy groups in CUR is involved in this hydrogen bond (with Lys163) (Table 3 and Fig 10).

**Table 3:** ChemPLP scores and hydrogen bond distances for curcumin derivatives from docking into *T*. *cruzi* tubulin.

Ligand	ChemPLP score	Hydrogen bond distances (Å)			
		Glu69	Lys163	Ser165	His396
CUR	51.56	2.90	2.83	2.35	2.88
DMC	50.06	2.99	2.63	2.43	2.92
BDMC	46.49	2.96	-	2.57	3.05

## Conclusions

The results obtained herein revealed natural curcumin as a hit compound for the development of new effective chemotherapeutic agents for the treatment of *Trypanosoma cruzi* infections. Selective toxic effects were observed for curcumin as well as for the two other minor natural diarylheptanoids present in turmeric, demethoxycurcumin and bisdemethoxycrucumin, against epimastigote forms of *T*. *cruzi*. The three compounds showed inhibitory effects onthe release of trypomastigotes from infected macrophages *in vitro*. These compounds had no cytotoxic activity against two different murine cell types, indicating a selective toxicity against *T*. *cruzi* cells. The inactivity of dihydropyranone derivative cyclocurcumin indicates the importance of diarylheptanoid scaffold for the trypanocidal activity. Electron microscopy studies showed significant ultrastructural changes in parasite cells pointing out the disarray of the cytoskeleton as the probable mechanism of action, which does not appear to be associated with the interference in the sterol synthesis as these compounds did not inhibit *T*. *cruzi* 14α-demethylase (CYP51), and no changes were observed in the ratio between ergosterol/lanosterol from *T*. *cruzi* epimastigotes treated with curcumin. The presence of multiple flagella and the uncompleted cell division observed in treated *T*. *cruzi* epimastigotes may be related to impaired cytoskeleton functioning in cytokinesis process, possibly related to tubulin polymerization process, as well as basal body division. The flow cytometry analysis of DNA content of the parasites showed that their treatments with curcuminoids resulted in an accumulation of cells in the G_2_/M phase, with consequent reduction of cells in G_1_ phase. This set of experimental observations is consistent with the action of microtubule-interacting agents as paclitaxel, used as positive control. Accordingly, the effects of curcuminoids in epimastigotes cells cycle can be correlated with the results highlighted in electron microscopy experiments.

Docking studies with a *T*. *cruzi* tubulin β-α-β trimer model showed that the three natural diarylheptanoid (CUR, DMC and BDMC) are able to effectively interact with curcumin binding site previously described in the literature, presenting an interaction profile that may explain the differences in the experimentally observed activity for each derivative. These results explain the effects of the three diarylheptanoids against proliferative epimastigote and amastigote forms of *T*. *cruzi*. The effect against epimastigotes was directly observed and for amastigotes it was indirectly assessed by the strong inhibitory effect of diarylheptanoids on the release of trypomastigotes from host infected cells. Other mechanisms of action, however, cannot be excluded and further studies are necessary.

## Materials and Methods

### Ethics Statement

This study was carried out in strict accordance with the recommendations in the Guide for the Care and Use of Laboratory Animals of the National Institutes of Health (USA). The protocol was approved by the Committee on the Ethics of Animal Experiments of the Health Science Center of the Federal University of Rio de Janeiro (CEUA-CCS, Permit Number: IMPPG 038/16) and all efforts were made to minimize suffering.

### Chemistry

Melting points were determined on a Büchi B-510 apparatus and are uncorrected. The ^1^H-NMR (500 MHz) and ^13^C-NMR (125 MHz) spectra were recorded on a Bruker UltrashieldPlus Spectrometer (BrukerBioSpin GmbH, Rheinstetten, Germany) operating at 500 MHz for ^1^H and 125 MHz for ^13^C. ^1^H and ^13^C-NMR shifts (δ) are reported in parts per million (ppm) with respect to DMSO-*d*_*6*_ (2.50 ppm for ^1^H and 39.7 ppm for ^13^C). Chemical shifts (δ) were reported in ppm and coupling constants (J) in Hertz [Hz]. Signal multiplicity was assigned as singlet (s), doublet (d), doublet of doublets (dd), triplet (t), quartet (q), multiplet (m) and broad signal (bs). All pectroscopic data are available in S1 File. All the reactions involving microwave instrumentation used the Discover SP system (CEM Inc., Matthews, NC, USA) and were performed in open vessel mode. Analytical thin-layer chromatography (TLC) was performed on precoated silica gel plates (0.25 mm layer thickness) in an appropriate solvent and the spots were visualized under UV light (254 nm and 365 nm).

#### Isolation of curcuminoids from commercial source

2g of commercial curcumin purchased from Aldrich^®^ were dissolved in 200 mL of methanol at heating. After complete solubilization, 50 mL of distilled water were added. The solution was filtered and after cooling, the precipitate formed was collected by vacuum filtration. After successive recrystallizations, 1.2 g of pure curcumin were collected as an orange amorphous solid (60% yield). m.p.: 182–185°C (Lit.: 183°C)[6]. ^1^H-NMR δ (ppm): 9.70 (s, 2H, -OH), 7.57 (d, 2H, *J* = 15 Hz), 7.35 (s, 2H), 7.18 (d, 2H, *J* = 10 Hz), 6.86–6.77 (m, 4H), 6.08 (s, 1H), 3,85 (s, 6H, -OCH_3_) (Fig A in S1 File); ^13^C NMR δ (ppm): 183.72, 149.84, 148.46, 141.22, 126.82, 123.64, 121.57, 116.19, 111.80, 101.37, 56.16 (Fig B in S1 File). EM/HR (369.1336) (Fig C in S1 File).

The mother liquor from curcumin recrystallization was submitted to vacuum until complete removal of methanol, resulting in 0.72 g of an orange solid, which was used for the isolation of the curcuminoids through a silica column. 100 g of silica gem 70–200 mesh were suspended in 100 mL distilled water. 5 g of NaH_2_PO_4_ were solubilized on this suspension. The system was stirred for 30 minutes at room temperature. Then were added 100 mL of acetone. After 10 minutes of stirring, the silica was filtered and kept in an oven at 200°C overnight. The silica was then employed in a chromatographic column used to isolate the natural curcuminoids, using dichloromethane as eluent. The fractions were grouped according to their TLC profile and after the removal of the solvent, the curcuminoids could be obtained in suitable purities. Demethoxycurcumin (DMC) was isolated as an amorphous orange solid in 14.25% yield. m. p. 179–183°C (Lit.: 181–182°C)[6]. ^1^H-NMR δ (ppm): 10.08 (bs, 1H, -OH), 9.72 (bs, 1H, -OH), 7.61–7.56 (m, 4H), 7.36 (s, 1H), 7.17 (s, 1H) 6.86–6.71 (m, 5H), 6.07 (s, 1H), 3,87 (s, 3H, -OCH_3_) (Fig D in S1 File); ^13^C NMR δ (ppm): 183.78, 183.65, 160.31, 149.85, 148.49, 141.22, 140.88, 130.86, 123.72, 121.52, 121.31, 116.41, 116.17, 111.70, 101.46, 56.16 (Fig E in S1 File); EM/HR (339.1230) (Fig F in S1 File). Bisdemethoxycurcumin (BDMC) was isolated as a reddish solid in 3.0% yield. m.p. 230–233°C. (Lit.: 232–234)[6]. ^1^H-NMR δ (ppm): 10.09 (s, 2H, -OH), 7.58–7.54 (m, 4H), 6.83 (d, 4H, 8Hz), 6.71 (d, 2H, *J* = 18 Hz), 6.05 (s, 1H) (Fig G in S1 File); ^13^C NMR δ (ppm): 183.68, 160.28, 140.84, 130.82, 126.27, 121.23, 116.37, 101.45 (Fig H in S1 File); EM/HR (309.1124) (Fig I in S1 File).

#### General procedure for the synthesis of symmetrical curcuminoids

In a round bottom flask with 10 mL capacity containing a magnet, 2,4-pentonedione (1 g, 0.1 mol) and boric anhydride (696 mg, 0.1 mol) were added, followed by catalytic amounts of acetic acid and morpholine. Then were added two equivalents of appropriate aldehyde and the flask was submitted to microwave irradiation (300 W) for 5 minutes in the open vessel mode. After the aldehyde were consumed (monitored by TLC), the oil formed was solubilized with aid of ultrasound in 5 mL of methanol. After the formation of an intense red solution, 5 mL of DMSO were added and the flask was connected to a reflux apparatus and heated at 100°C for 1h. Addition of cold water gives an orange solid which was separated by vacuum filtration on a Buchner funnel and purified by column chromathography on silica using suitable eluent.

#### Procedure for the cyclocurcumin synthesis

In a round bottom flask with 50 mL capacity, 100 mg of purified curcumin was suspended in 20 mL of dry benzene. 0.6 mL of trifluoracetic acid was added and the system was stirred at room temperature and protected from light for 72h. The mixture was concentrated under vacuum and subjected to chromatograph. 21mg (21% yield) of cyclocurcumin (CC) was isolated as a yellow oil. ^1^H-NMR δ (ppm): 7.32 (d, 1H, *J* = 10 Hz), 7.06 (d, 1H, *J =* 5 Hz), 7.02 (m, 4H), 6.93 (d, 1H, *J =* 10 Hz), 6.47 (d, 1H, *J* = 15Hz) 5.86 (s, 1H), 5.78 (s, 1H), 5.62 (s, 1H), 5.41 (dd, 1H, *J*_*1*_ *=* 15Hz, *J*_2_ = 5Hz), 3.98 (s, 3H), 3.96 (s, 3H), 2.96 (dd, 1H, *J*_*1*_ = 15Hz, *J*_*2*_ = 10Hz), 2.68 (dd, 1H, *J*_*1*_ = 15Hz, *J*2 = 5Hz) (Fig J in S1 File); ^13^C NMR δ (ppm): 193.07, 168.90, 147.54, 146.61, 146.70, 146.23, 137.67, 130.38, 127.76, 122.52, 119.91, 118.93, 114.79, 114.55, 109.11, 109.00, 105.56, 80.88, 56.07, 56.00, 43.25 (Fig K in S1 File); EM/HR (369.1337) (Fig L in S1 File).

### Biological Assays

#### Parasites

Epimastigotes of *T*. *cruzi* (Dm28c strain) were cultured at 27°C in Bacto^™^ Brain Heart infusion (BHI, Becton Dickinson Company, USA) supplemented with 10 μg.mL^-1^ hemin, 0.02 g.L^-1^ folic acid (from Sigma-Aldrich, USA) and 10% (v/v) of heat inactivated fetal bovine serum (FCS, Gibco/Life technologies).

#### Anti-epimastigote effect

Epimastigotes forms of *T*. *cruzi*, Dm28c strain (2x10^5^ cells.mL^-1^), were grown for 7 days in BHI culture medium at 26°C. Some cultures were treated with curcumin (CUR), demethoxycurcumin (DMC), bisdemethoxycurcumin (BDMC) or cyclocurcumin (CC) at concentrations of 100, 50, 25 and 2 μM. Viable forms were counted in Neubauer chambers under phase contrast microscopy using 40X objectives. Data representing three independent experiments.

#### Cytotoxic effect on murine macrophages

Macrophages harvested from the peritoneal cavity of BALB/c mice were plated at a concentration of 5x10^5^ cells.mL^-1^ in triplicates. Cells were treated for 48 h with the indicated doses of CUR, DMC, BDMC and CC ranging from 2 to 100 μM. Cell viability was assessed by the dye exclusion method using trypan blue and the results show data representative of three independent experiments.

#### Cytotoxic effect on murine splenocytes

Splenic lymphocytes were obtained from male BALB/c mice and plated at 5x10^5^ cells.mL^-1^ in triplicate. Cells were treated for 48h with the indicated concentrations of CUR, DMC, BDMC and CC ranging from 2 to 100 μM. Viability was evaluated by the exclusion method using trypan blue and the data shown is representative of three independent experiments.

#### Inhibition of trypomastigotes release of *T*. *cruzi* in vitro

2.10^4^ murine peritoneal macrophages were infected with 10^5^ (5 parasites per host cell) chemically-induced metacyclic forms of *Trypanosoma cruzi* (Dm28c strain) obtained as described [49]. Some cultures were treated with Benznidazole, CUR, DMC, BDMC and CC at the concentration of 100 μM. After 7 days the number of trypomastigotes released into the supernatant of cultured were determined. The cultures were made in triplicate and the data are representative of three independent experiments. The culture were compared using the T-Student test unpaired (Prism Graph Pad) p* > 0.05.

#### Transmission electron microscopy

Epimastigotes of *Trypanosoma cruzi*, Dm28c strain, were treated with curcuminoids at its IC_50_ concentrations (CUR 10.13 μM, DMC 11.07 μM and BDMC 45.33 μM) and maintained in brain heart infusion (BHI) supplemented with 10% fetal bovine serum at 25°C (±1°C). The protozoa were washed in PBS and fixed in Karnovsky for 24 h. Next, the samples were post-fixed in 1% osmium tetroxide, 0.8% potassium ferricyanide and 5 mM CaCl_2_ sheltered from light for 40 minutes at room temperature and then dehydrated in increasing concentrations of acetone (15–100%). The samples were embedded in epoxy Polybed (Polysciences, Inc) resin. After 72 hours at 60°C, the samples were sectioned with ultramicrotome (Reichert, Leica), contrasted in aqueous solutions of 2% uranyl acetate for 20 minutes and 1% lead citrate for 5 minutes and observed under a transmission electron microscope (Zeiss EM 109 a 80 kV) [19].

#### Scanning electron microscopy

Parasites were fixed as described above and adhered to coverslips previously coated with a solution of poly-*L*-lysine. Next, dehydration was performed in series of increasing acetone concentrations (15–100%). The samples were subjected to critical point drying and metallized with an approximately 20 nm-thick gold layer to be observed in a scanning electron microscope JEOL model JEOL [19].

#### Sterol extraction

6x10^6^ epimastigotes of *T*. *cruzi* (Dm28c strain) were grown for 7 days in 30mL of BHI culture medium at 26°C. Some cultures were treated with curcumin (CUR, 5 and 10 μM), posaconazole (0.05 and 0.1 μM). The cultures were submitted to centrifugation at 2100 rpm for 10 minutes. The supernatants were discarded and the pellets with *T*. *cruzi* cells were submitted to sterol extraction. The wet pellets of *T*. *cruzi* cells (wild and treated with curcumin) were adjusted to 100 mg, then 3 mL of alcoholic potassium hydroxide solution (25% m/v) was added, followed by a vigorous agitation for 1 minute. The cell suspensions were incubated in a hot plate at 80°C during 1 hour. The tubes were cooled to room temperature. Sterols were extracted by addition of 1 mL of water and 3 mL of *n*-hexane in each tube, followed by a vigorous agitation for exactly 3 minutes. The organic phase was transferred to a glass tube and the *n*-hexane was removed to dryness under vacuum. The extracted sterols were dissolved in spectroscopic grade *n*-hexane prior to HPLC analysis [40].

#### Sterol analysis

Analysis of the isolated lipid fractions of *T*. *cruzi* epimastigotes were carried out in an HPLC Shimadzu (LC-20AT), oven CTO 20A; Detector PDA (SPD-M20A), auto injector Sil-10AF and controller CBM-20A. The method used was the reverse phase (C18-column Betasil-THERMO, 25 cm x 4.6 mm x 5 μm) at isocratic mode, using methanol (98%) and acetonitrile (2%) as eluent, at a flow of 1.1 mL.min^-1^. The detection was carried out at 280 nm (to ergosterol) and 243 nm (to lanosterol). The injection volume was 20 μL and the oven temperature was 27°C. The steroids identification (ergosterol e lanosterol) were made through comparison with the retention times and UV-curves with commercially-available steroids (Sigma-Aldrich).

#### Flow cytometry analysis of the cell cycle

Epismatigotes forms of *T*. *cruzi* (Dm28c strain) were grown at 28°C in BHI medium at a concentration of 2x10^6^ cells.mL^-1^ in 12-well culture plates (TPP^™^, Switzerland). Test compounds (CUR, DMC and BMDC) were added in triplicate at concentrations of 10, 50 and 100 μM. The controls used were the vehicle (DMSO 0.02%) and paclitaxel at 20 μM concentration. After 48 h of incubation, parasites were fixed in ethanol 70% for 30 min at -20°C. Next, cells were centrifuged at 200 rcf for 8 min and washed with PBS. Then, the samples were incubated for 10 min with a permeabilization buffer (0.2 M Na_2_HPO_4_ and 0.005% Triton-X, pH 7.8). Finally, cells were centrifuged at 200 rcf for 8 min and stained in a solution of 50 μg.mL^-1^ propidium iodide (PI) and 0.2 μg.mL^-1^ RNAse for 30 min at room temperature. DNA content was analyzed measuring PI fluorescence in a BD Accuri C5 cytometer (Becton Dickinson Company, USA) at 488 nm excitation wavelength and 585/40 nm emission filter.

#### Statistical analysis

Statistical analyses were performed with GraphPad Prism 4 software, using one-way ANOVA test. Results were expressed as mean ± standard error (S.E.). Differences between control and treated group were considered statistically significant when *P*≤0.05.

### Molecular Modeling

Based on the experimental results that are indicative of tubulin as a possible target for the active compounds, we implemented a molecular docking study with *T*. *cruzi* tubulin to identify possible molecular reasons for the difference in activities observed for CUR, DMC and BDMC. Ligands that are well-known to disturb tubulin-microtubule dynamics are colchicine, vinblastine and taxol, for which specific binding sites were identified in tubulin. Jackson and collaborators have shown that CUR also binds to mammalian tubulin, inducing mitotic catastrophe, and impeding normal endothelial cell proliferation [50]. A large number of drugs are known to inhibit tubulin by binding at one of the three characterised tubulin ligand sites—taxol, colchicine and vinca-binding sites. Due to its action, it would be possible for CUR to bind at the colchicine or vinca-binding sites, but Chakraborti *et al*. [33], using fluorescence spectroscopy with tubulin isolated from goat brains, showed that CUR binds to a fourth binding site located 32 Å away from the colchicine-binding site.

There is no available crystallographic structure for *T*. *cruzi* tubulin, so it was necessary for the docking study the construction of a 3D model. Microtubules are composed of two globular protein subunits, *α*- and *β*-tubulin, that form an *α*,*β*-heteropolymer. There are sequences for both *T*. *cruzi* α- and β-tubulin obtainable in the UniProtKB/Swiss-Prot protein sequence database (Q27352 for the α-tubulin and P08562 for β-tubulin), which we used separately for construction of homology models with the automated mode of the protein structure homology-modeling server, Swiss-Model [51]. In order to obtain complete binding sites located at the interfaces between *α*- and *β*-tubulin subunits and also between *β*- and *α*-tubulin subunits, a *β-α-β-*heterotrimer was constructed with the modelled subunits.

For the three possible binding sites for CUR and its derivatives, there are crystallographic structures of tubulin with ligands co-crystallized in the colchicine and vinca-binding sites. As a previous test for the ability of the docking program GOLD 5.2 (CCDC Software Ltd., Cambridge, UK) to find reliable solutions for the docking into tubulin, we implemented redocking experiments at both sites. All of the scoring functions available in the program were tested through redocking procedures with the PDB crystallographic structures 4EB6 (*Ovisaries* tubulin co-crystallized with vinblastine) and 4O2B (*Bostaurus* tubulin co-crystallized with colchicine). Hydrogen atoms were added to proteins structures based on ionization and tautomeric states defined by the program. To allow the best orientation of hydrogen bonds involving the serine, threonine, tyrosine and lysine side chains, they were set free to rotate during the docking procedure. In the course of the searching procedure, 100,000 genetic operations (crossover, migration, mutation) were used for each docking run. Radius of binding sites for the enzymes was set up to 10 Å around atoms from adequate amino acids selected based on literature information for each binding site.

Spartan’14 program [Wavefunction, Inc.] was utilised to construct and optimize CUR, DMC and BDMC structures with the PM3 method [52]. Several poses were obtained for each compound in all proteins, and the best-ranked pose for each one was chosen for analysis of the interactions with the amino acid residues. In the GOLD docking program, the docking functions yield fitness scores, which are dimensionless values. In each case, the score values are a guide of how good the docking pose is, with a higher score indicating a better docking result.

## Supporting Information

S1 Fig TLC and HPLC analysis of commercial curcumin. A) TLC using hexanes/ethyl acetate 50% as eluent. B) TLC using methanol/dichloromethane 2% as eluent. C) HPLC analysis at reversed-phase column C18 using acetonitrile/water as eluent.
(TIF)

S2 Fig *T*. *cruzi* epimastigotes (Dm28c strain) were cultivated for 7 days in BHI (Brain Heart Infusion) culture medium supplemented with hemin, folic acid and 10% fetal bovine serum at 26°C, and readily treated with CUR, DMC, BDMC and CC at the indicated concentrations ranging from 2 to 100μM. Viable forms were counted on Neubauer chambers under phase microscopy at the seventh day. The data presented were obtained from three representative independent experiments.
(TIF)

S3 Fig Peritoneal macrophages obtained from BALB/c mice were cultured in triplicates at concentration of 5x10^5^cells.mL^-1^. The cells were treated for 48 hours with different concentrations of CUR, DMC, BDMC and CC, ranging from 2 to 100μM. Cell viability was assessed by exclusion method using trypan blue. The results shown were obtained from representative data of three independent experiments.
(TIF)

S4 Fig Lymphocytes were obtained from the spleen of BALB/c mice and cultivated at concentration of 5x105 cells.mL^-1^. The cells were treated for 48 hours with CUR, DMC, BDMC and CC at indicated concentrations (2 to 100 μM). Cell viability was assessed by exclusion method using trypan blue. The results shown were obtained from representative data of three independent experiments.
(TIF)

S5 Fig Representative HPLC chromatograms of unsaponified lipds from *T*. *cruzi* epimastigotes. A) Negative control: epimastigotes cultivated, without treatment; B) Solvent control: epimastigotes treated with DMSO 0.2%; C) Positive control: epimastigotes treated with posaconazole 0.05 μM; D) Treatment: epimastigotes treated with curcumin 10.0 μM.
(TIF)

S6 Fig Representative fluorescence histograms showing DNA content of cell-cycle phases (Sub-G_1_, G_1_, S and G_2_) of parasites (Dm28c strain). A) Negative control (DMSO 0.02%); B) Positive control (paclitaxel 20μM); C) Treatment with 100 μM BDMC; D) Treatment with 10 μM CUR; E) Treatment with 50 μM CUR; F) Treatment with 100 μM CUR; G) Treatment with 10 μM DMC; H) Treatment with 50 μM DMC; I) Treatment with 100 μM DMC. The data shown were obtained from three independent experiments.
(TIF)

S1 File Spectral data (^1^H, ^13^C, HRMS) of CUR, DMC, BDMC and CC. (PDF)
