# Supplementary figures and images for: The Effectiveness of Natural Diarylheptanoids against Trypanosoma cruzi: Cytotoxicity, Ultrastructural Alterations and Molecular Modeling Studies
Source: PLoS One. 2016 Sep 22;11(9):e0162926. doi: 10.1371/journal.pone.0162926 (PMC5033595; doi:10.1371/journal.pone.0162926)

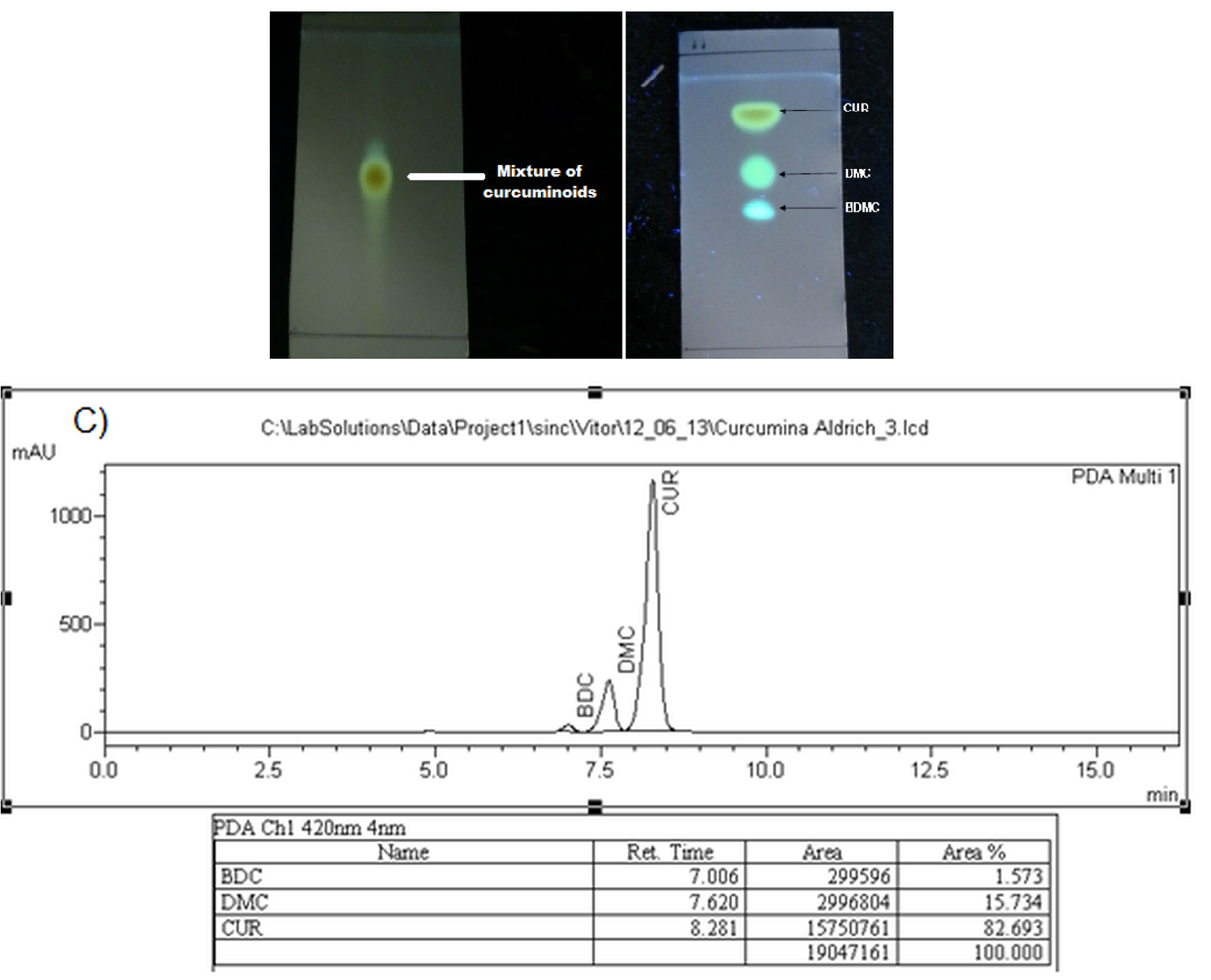

Supplement: S1 Fig — A) TLC using hexanes/ethyl acetate 50% as eluent. B) TLC using methanol/dichloromethane 2% as eluent. C) HPLC analysis at reversed-phase column C18 using acetonitrile/water as eluent. (TIF) [file pone.0162926.s001.tif]

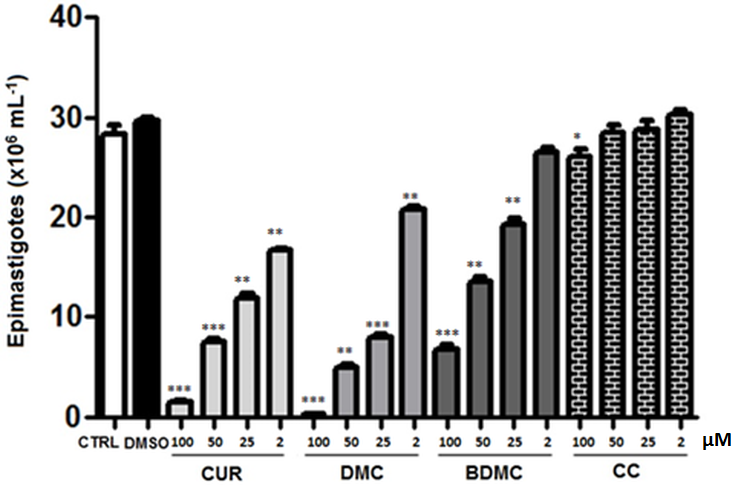

Supplement: S2 Fig — Viable forms were counted on Neubauer chambers under phase microscopy at the seventh day. The data presented were obtained from three representative independent experiments. (TIF) [file pone.0162926.s002.tif]

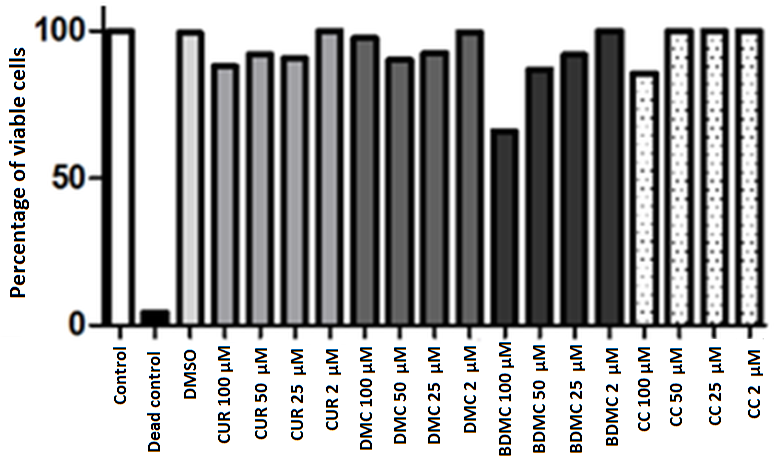

Supplement: S3 Fig — The cells were treated for 48 hours with different concentrations of CUR, DMC, BDMC and CC, ranging from 2 to 100μM. Cell viability was assessed by exclusion method using trypan blue. The results shown were obtained from representative data of three independent experiments. (TIF) [file pone.0162926.s003.tif]

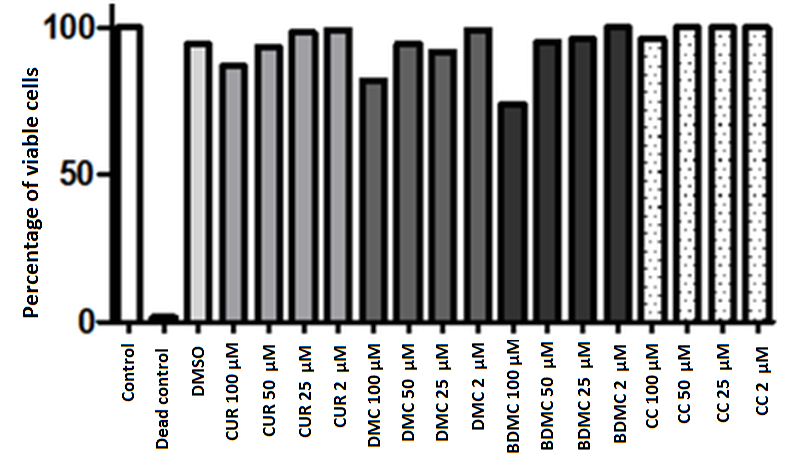

Supplement: S4 Fig — The cells were treated for 48 hours with CUR, DMC, BDMC and CC at indicated concentrations (2 to 100 μM). Cell viability was assessed by exclusion method using trypan blue. The results shown were obtained from representative data of three independent experiments. (TIF) [file pone.0162926.s004.tif]

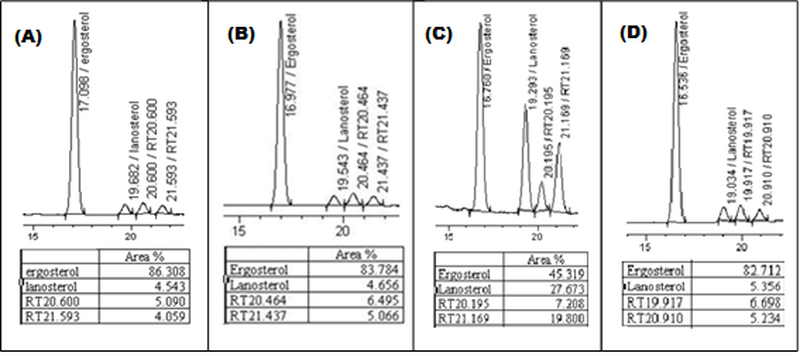

Supplement: S5 Fig — A) Negative control: epimastigotes cultivated, without treatment; B) Solvent control: epimastigotes treated with DMSO 0.2%; C) Positive control: epimastigotes treated with posaconazole 0.05 μM; D) Treatment: epimastigotes treated with curcumin 10.0 μM. (TIF) [file pone.0162926.s005.tif]

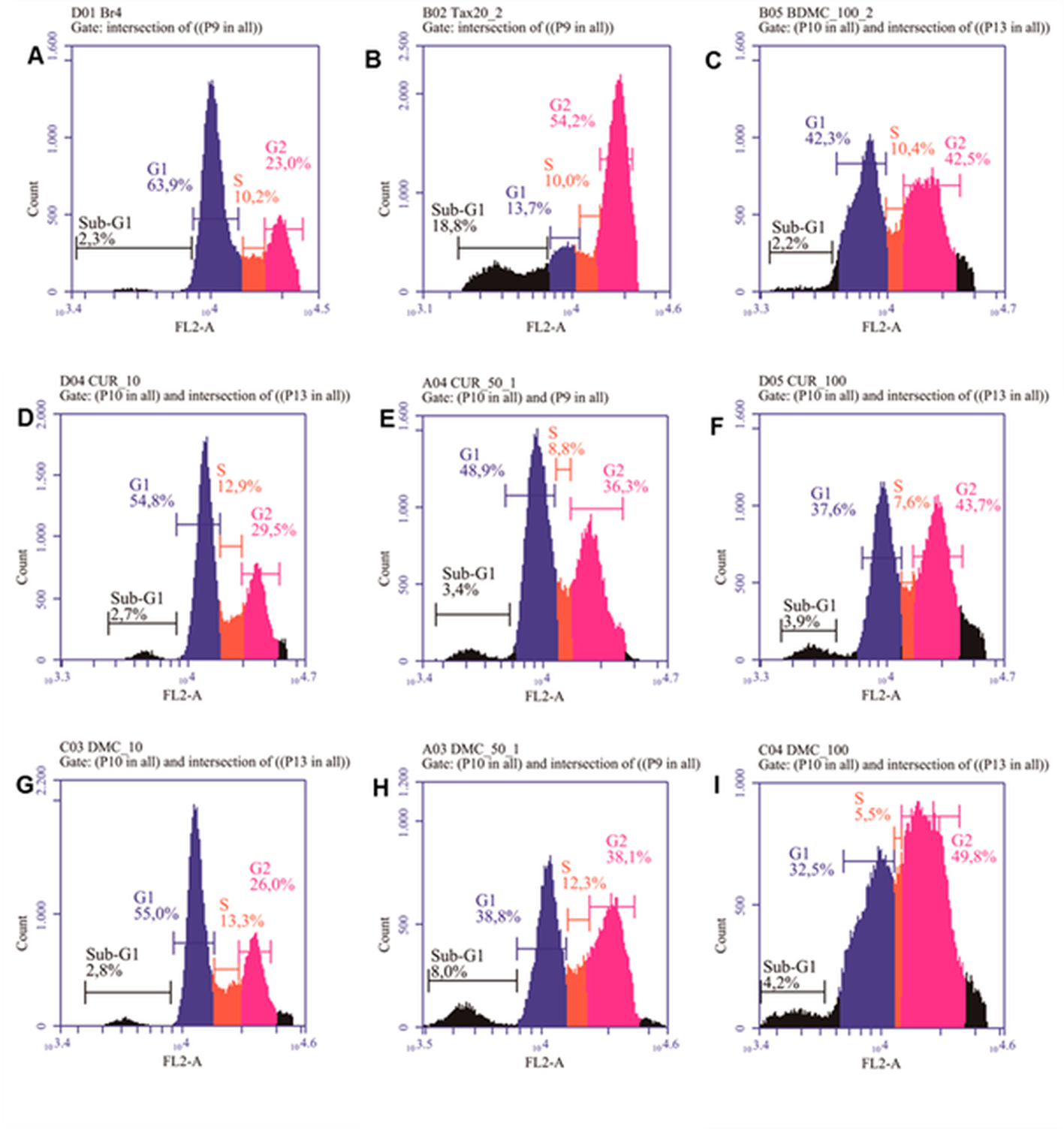

Supplement: S6 Fig — A) Negative control (DMSO 0.02%); B) Positive control (paclitaxel 20μM); C) Treatment with 100 μM BDMC; D) Treatment with 10 μM CUR; E) Treatment with 50 μM CUR; F) Treatment with 100 μM CUR; G) Treatment with 10 μM DMC; H) Treatment with 50 μM DMC; I) Treatment with 100 μM DMC. The data shown were obtained from three independent experiments. (TIF) [file pone.0162926.s006.tif]
