# Supplementary material for: The Effectiveness of Natural Diarylheptanoids against Trypanosoma cruzi: Cytotoxicity, Ultrastructural Alterations and Molecular Modeling Studies
Source: PLoS One. 2016 Sep 22;11(9):e0162926. doi: 10.1371/journal.pone.0162926 (PMC5033595; doi:10.1371/journal.pone.0162926)

S1_File

Spectral data of CUR, DMC, BDMC and CC

Figure A: Chemical structure, NMR ^{1}H data and chemical shifts for Curcumin (CUR) in DMSO-d_6 at 500 MHz

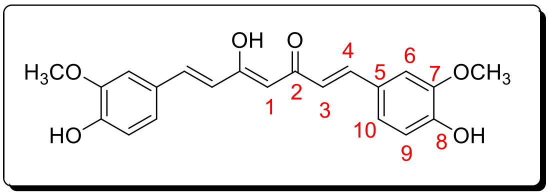

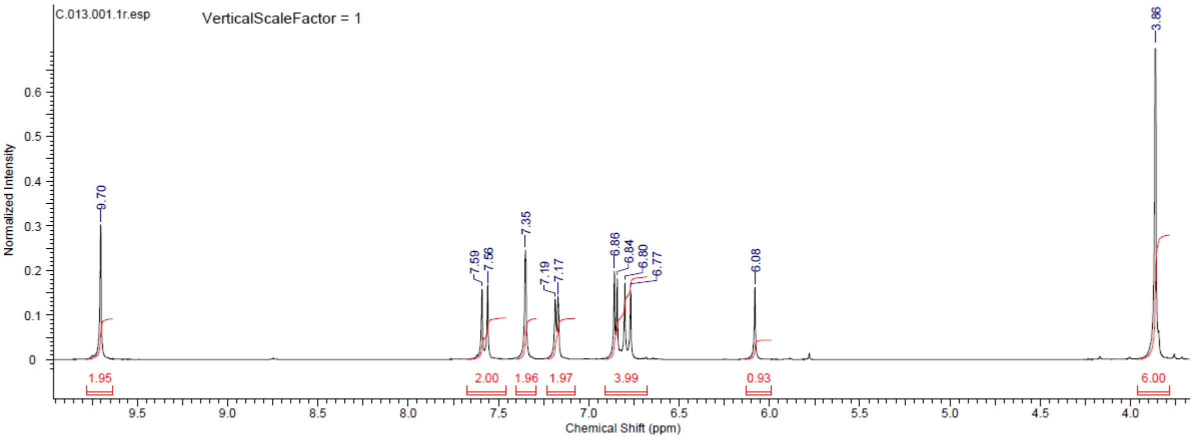

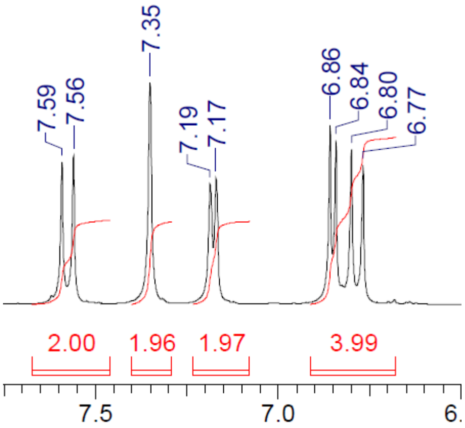

Proton	δ (ppm)	m	Integration	*J* (Hz)
1	6.08	s	1	-
3	6.78	d	2	15
4	7.57	d	2	15
6	7.35	s	2	-
9	6.85	d	2	10
10	7.18	d	2	10
Ar-OH	9.70	s	2	-
Ar-OCH_3	3.86	s	6	-

Figure B: Chemical structure, NMR ^{13}C data and chemical shifts for Curcumin (CUR) in DMSO-d_6 at 125 MHz

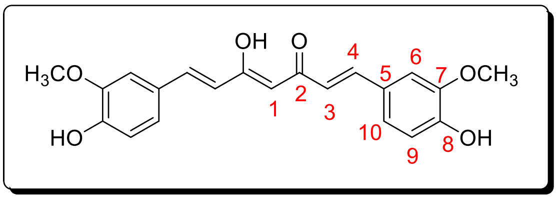

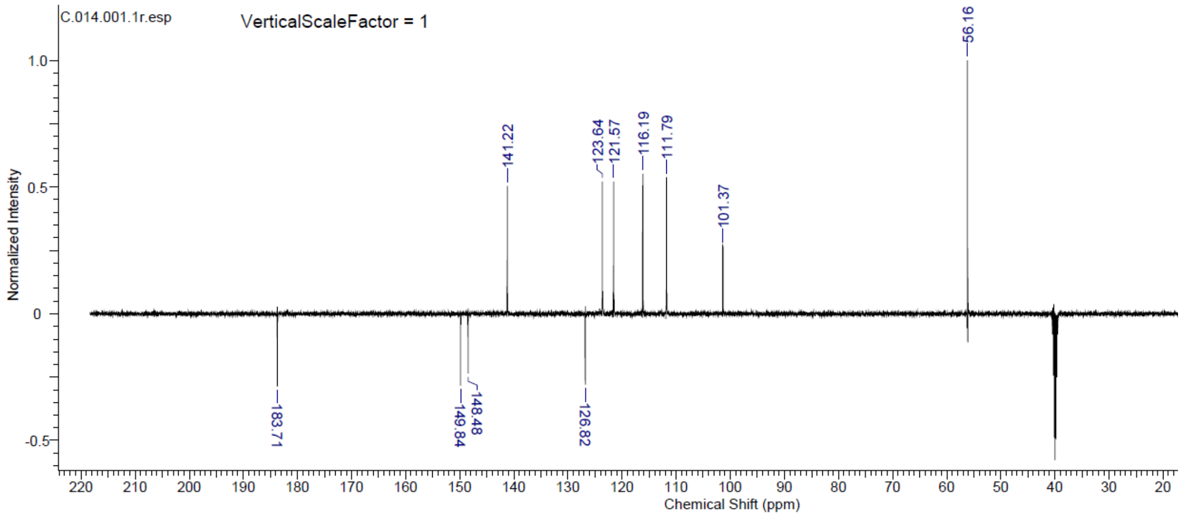

Carbon	δ (ppm)	m
1	101.37	CH
2	183.71	C
3	123.64	CH
4	141.22	CH
5	126.82	C
6	111.79	CH
7	148.48	C
8	149.84	C
9	116.19	CH
10	121.57	CH
Ar-O$C\underline{H}_3$	56.16	CH_3

Figure C: HRMS for Curcumin (CUR)

$[M+H]^+$
Theoretical: 369.1332
Experimental: 369.1337
Error: 0.9ppm

CUR MS/MS

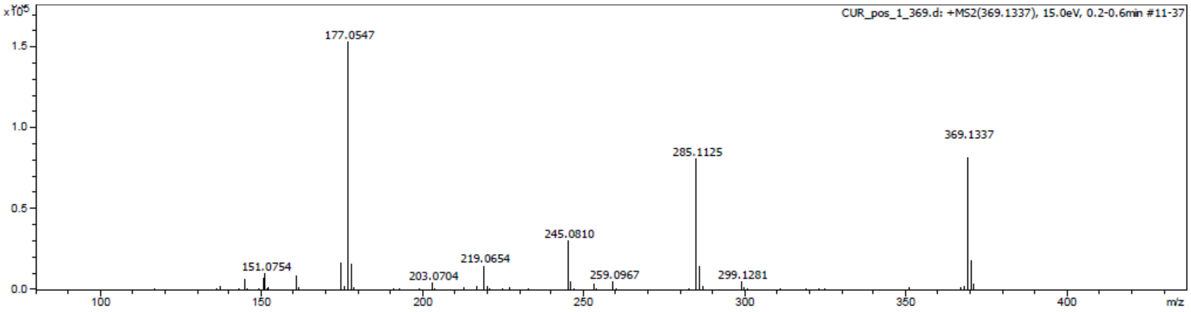

Figure D: Chemical structure, NMR ^{1}H data and chemical shifts for Demethoxycurcumin (DMC) in DMSO-d_6 at 500 MHz

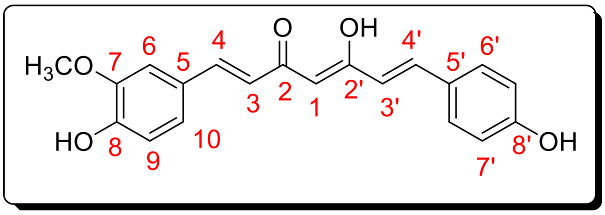

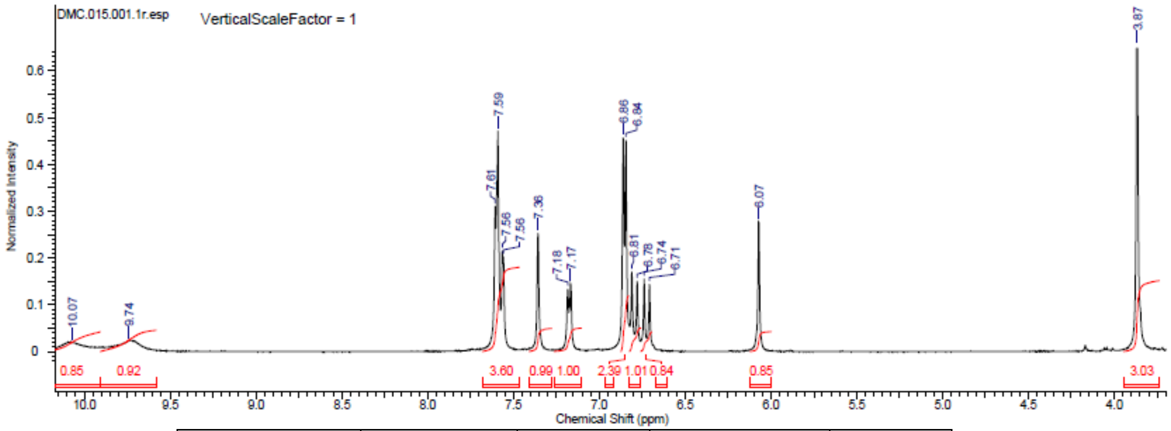

Proton	δ (ppm)	m	Integration	*J* (Hz)
1	6.07	s	1	-
3	6.78	d	1	15
3’	6.73	d	1	15
4	7.56	m	1	-
4’	7.60	m	1	-
6	7.36	s	1	-
6’	7.60	m	1	-
7’	6.85	d	1	10
9	6.85	d	1	10
10	7.18	d	1	10
Ar-O$\underline{H}$	9.74	sl	1	-
Ar’-OH	10.07	sl	1	
Ar-OC$\underline{H}_3$	3.87	s	3	-

Figure E: Chemical structure, NMR ^{13}C data and chemical shifts for Demethoxycurcumin (DMC) in DMSO-d_6 at 125 MHz

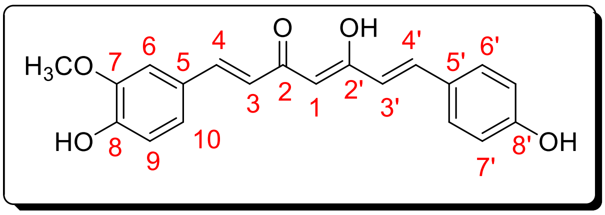

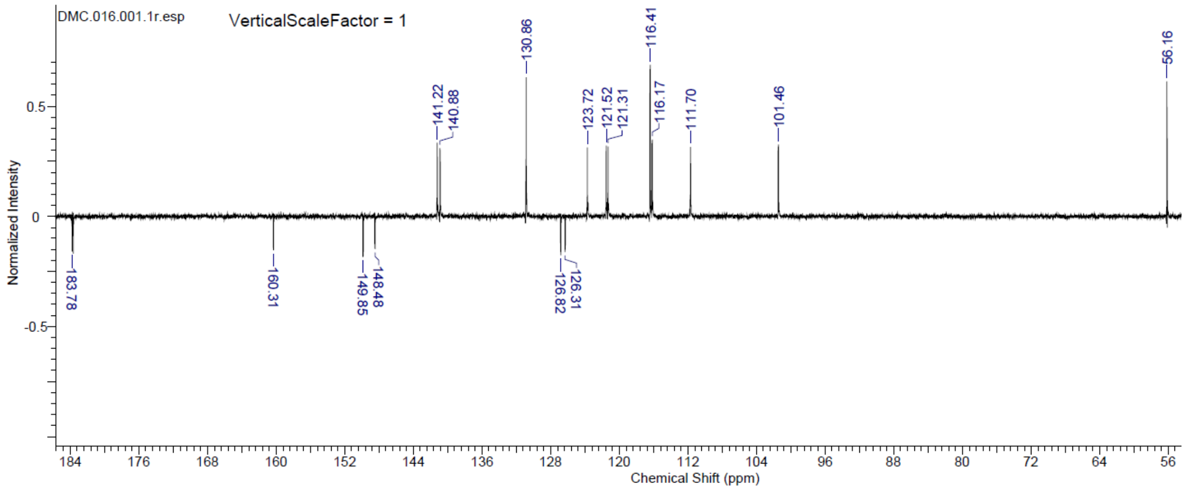

Carbon	δ (ppm)	m
1	101.37	CH
2	183.78	C
2’	184.07	C
3	123.72	CH
3’	121.31	CH
4	141.22	CH
4’	140.88	CH
5	126.82	C
5’	126.31	C
6	111.70	CH
6’	116.17	CH
7	148.48	C
7’	130.86	CH

Figure F: HRMS for Demethoxycurcumin (DMC)

[M+H]+
Theoretical: 339.1227
Experimental:
339.1234
Error: 1.0 ppm

DMC MS/MS

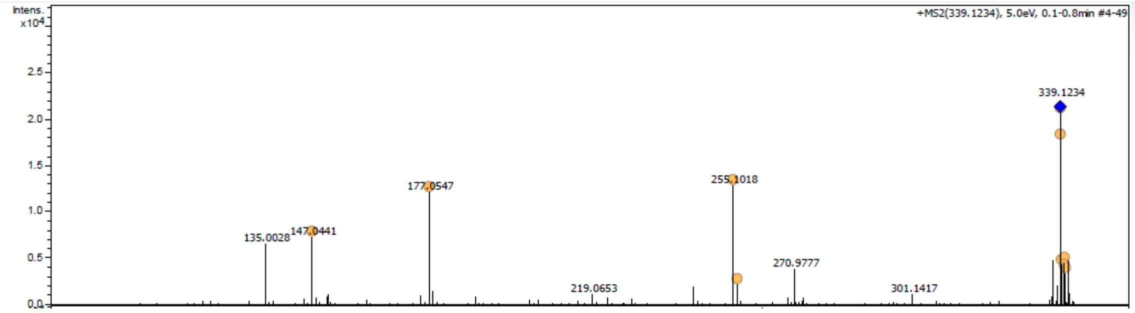

Figure G: Chemical structure, NMR ^{1}H data and chemical shifts for Bisdemethoxycurcumin (BDMC) in DMSO-d_6 at 500 MHz

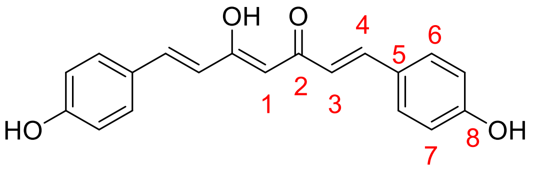

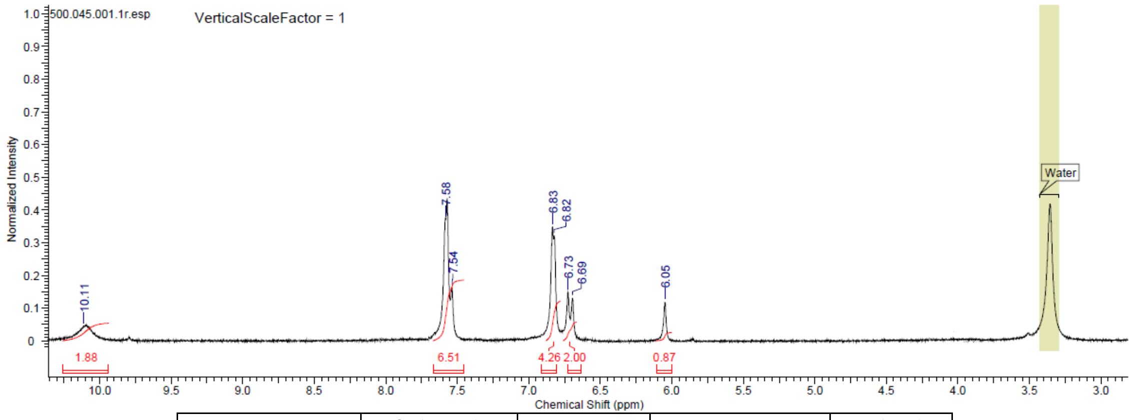

Proton	δ (ppm)	m	Integration	*J* (Hz)
1	6.05	s	1	-
3	6.71	d	2	20
4	7.54	m	-	-
6	7.58	m	-	-
7	6.83	d	4	10
Ar-OH	10.11	sl	2	-

Figure H: Chemical structure, NMR ^{13}C data and chemical shifts for Bisdemethoxycurcumin (BDMC) in DMSO-d_6 at 125 MHz

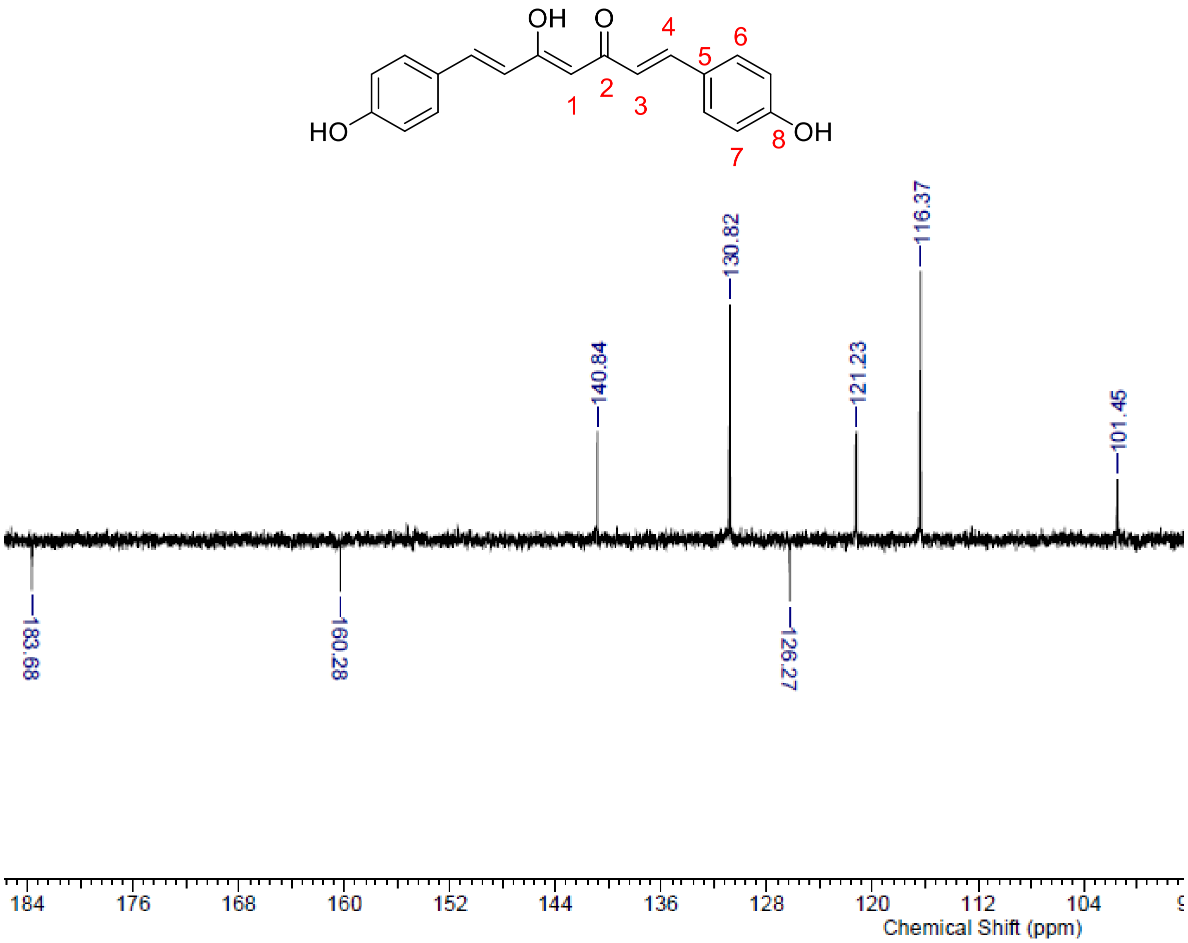

Carbon	δ (ppm)	m
1	101.45	CH
2	183.68	C
3	121.23	CH
4	140.84	CH
5	126.27	C
6	116.37	CH
7	130.82	CH
8	160.28	C

Figure I: HRMS for Bisdemethoxycurcumin (BDMC)

[M+H]+
Theoretical: 309.1121
Experimental: 309.1126
Error: -0.8ppm

BDMC MS/MS

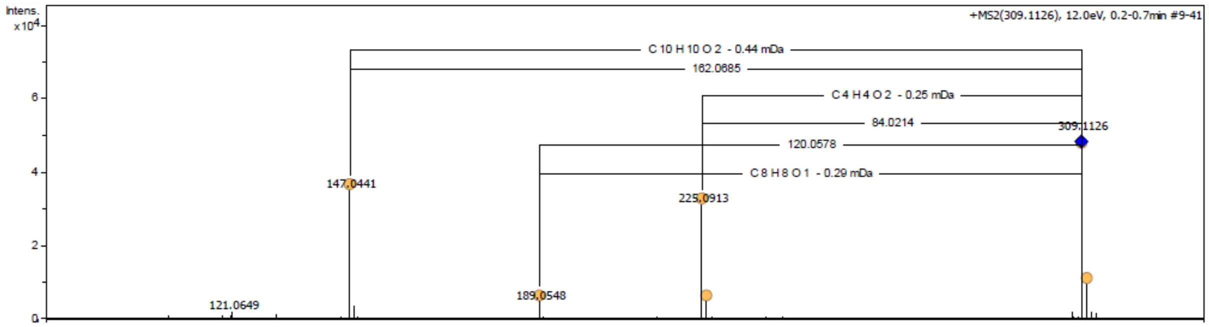

Figure J: Chemical structure, NMR ^{1}H data and chemical shifts for Cyclocurcumin (CC) in $CDCl_3$ at 500 MHz

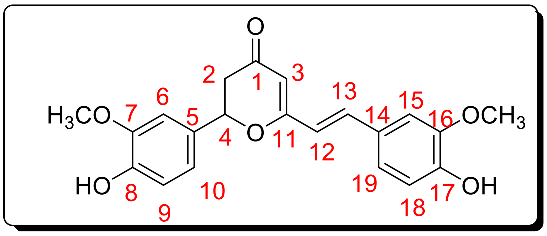

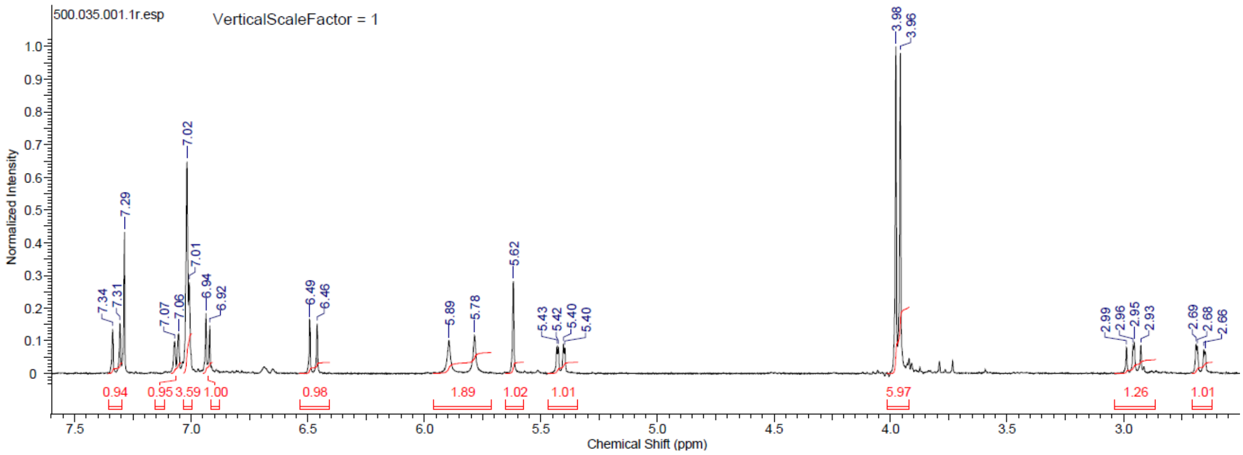

Proton	δ (ppm)	m	Integration	*J* (Hz)
2	2.96	dd	1	20/15
2’	2,86	dd	1	15/1
3	5.62	s	1	-
4	5.42	dd	1	15/1
6	7.02	m	1	-
9	7.06	d	1	10
10	6.93	d	1	10
12	6.47	d	1	15
13	7.32	d	1	15
15	7.02	m	1	-
18	7.02	m	1	-
19	7.02	m	1	-
Ar-OCH_3	3.98	s	3	-
Ar’-OCH_3	3.96	s	3	-
Ar-OH	5.89	s	1	-

Figure K: Chemical structure, NMR ^{13}C data and chemical shifts for Cyclocurcumin (CC) in $CDCl_3$ at 125 MHz

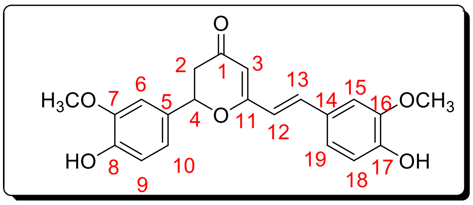

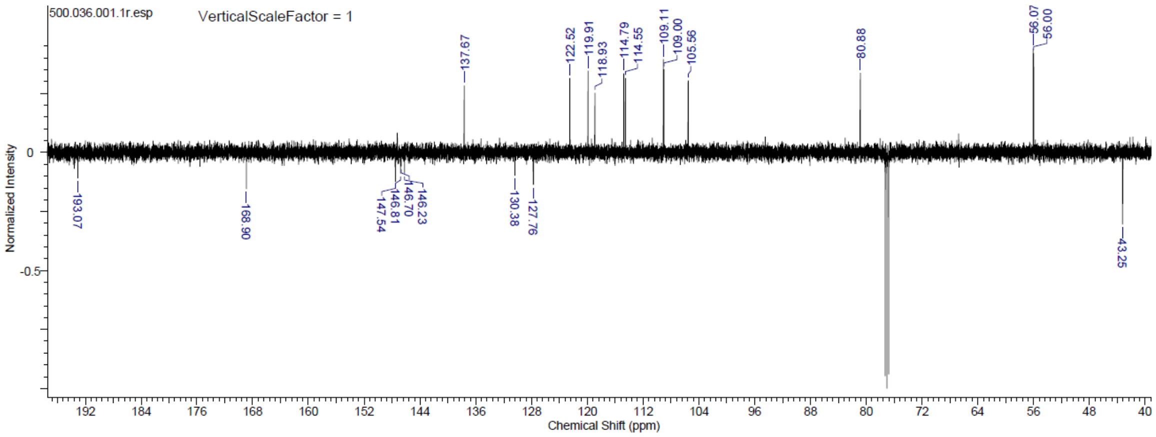

Carbon	δ (ppm)	m
1	193.07	C
2	43.25	CH_2
3	105.56	CH
4	80.88	CH
5	130.38	C
6	109.11	CH
7	146.70	C
8	146.23	C
9	114.55	CH
10	119.91	CH
11	168.90	C
12	118.93	CH
13	137.67	CH
14	127.76	C
15	109.00	CH
16	146.81	C
17	147.54	C
18	114.79	CH
19	122.52	CH
Ar-OCH_3	56.00	CH_3
Ar'-OCH_3	56.07	CH_3

Figure L: HRMS for Cyclocurcumin (CC)

[M+H]+
Theoretical: 369.1332
Experimental: 369.1337
Error: -1.2ppm

CC MS/MS

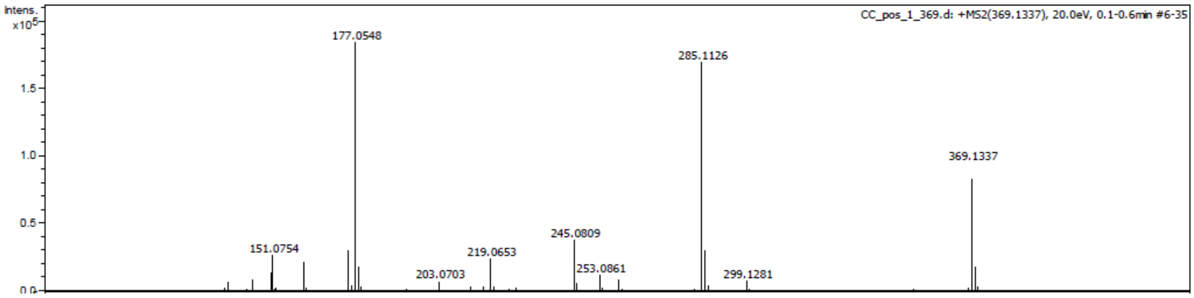

Supplement: S1 File — (PDF) [file pone.0162926.s007.pdf]
